# A Variable Order Fractional Differential-Based Texture Enhancement Algorithm with Application in Medical Imaging

**DOI:** 10.1371/journal.pone.0132952

**Published:** 2015-07-17

**Authors:** Qiang Yu, Viktor Vegh, Fawang Liu, Ian Turner

**Affiliations:** 1 The School of Mathematical Sciences, Queensland University of Technology, Brisbane, Queensland, Australia; 2 Centre for Advanced Imaging, University of Queensland, Brisbane, Queensland, Australia; Zhejiang University, CHINA

## Abstract

Texture enhancement is one of the most important techniques in digital image processing and plays an essential role in medical imaging since textures discriminate information. Most image texture enhancement techniques use classical integral order differential mask operators or fractional differential mask operators using fixed fractional order. These masks can produce excessive enhancement of low spatial frequency content, insufficient enhancement of large spatial frequency content, and retention of high spatial frequency noise. To improve upon existing approaches of texture enhancement, we derive an improved Variable Order Fractional Centered Difference (VOFCD) scheme which dynamically adjusts the fractional differential order instead of fixing it. The new VOFCD technique is based on the second order Riesz fractional differential operator using a Lagrange 3-point interpolation formula, for both grey scale and colour image enhancement. We then use this method to enhance photographs and a set of medical images related to patients with stroke and Parkinson’s disease. The experiments show that our improved fractional differential mask has a higher signal to noise ratio value than the other fractional differential mask operators. Based on the corresponding quantitative analysis we conclude that the new method offers a superior texture enhancement over existing methods.

## Introduction

Texture plays an important role in the identification of regions of interest in an image, hence texture enhancement is an essential component in digital image processing [1]. The aims of texture enhancement are to improve the overall visual effect of the image through purposefully emphasizing local or whole characteristic features and impair characteristics of little interest. The quality of the image, especially the texture, is critical in the clinical diagnosis and identification of pathology based on medical images.

Integral order differential mask operators, such as the Sobel, Roberts, Prewit and Laplacian techniques [1, 2], are widely used in image enhancement algorithms. However, there are several disadvantages using an integral order differential operator. For example, first order masks produce wide edges after image processing; second order masks generate double responses when the grey scale changes, and are therefore sensitive to noise [2, 3].

A growing number of research projects in science and engineering utilise fractional calculus to improve the understanding and description of physical phenomena [4–14]. These benefits provide an excellent motivation for researchers to apply fractional derivatives to digital image processing [3, 8, 15–27]. Chen et al. proposed a digital fractional order Savitzky–Golay differentiator, and experiments showed that it can estimate the fractional order derivative of the contaminated signal [15, 16]. Based on the Riemann–Liouville definition, Zhang et al. developed an algorithm to enhance the texture and edges of digital images [3]. Furthermore, the mask used had an improved visual effect with richer texture information than that obtained by integral order masks. Sejdić et al. showed that the fractional Fourier transform is potentially a very powerful tool in signal processing [17]. A stochastic fractal model was also developed for image processing, which is basically an analysis and synthesis tool for images [18]. Mathieu et al. applied fractional differential masks to detect edges, which improved the criterion of thin detection, or detection selectivity for parabolic luminance transitions [19]. Generally, robustness to noise was improved using these masks. Based on the Grünwald–Letnikov definition, Gao et al. applied a quaternion fractional differential to a colour image [23]. When applied to every channel of the image the experiments showed that their method, when compared to Sobel and mixed edge fractional techniques, has fewer false negatives in the textured regions and is better at detecting edges that are partially defined by texture. The use of an improved fractional differential operator based on a piecewise quaternion resulted in excellent textural detail enhancement of rich-grained digital images [21]. The authors subsequently proposed four new fractional directional differentiation masks and corresponding numerical simulation rules [22]. Experiments showed that their method can enhance the texture details of rich–grained digital images. Garg and Singh proposed a Grünwald–Letnikov fractional differential mask using a Lagrange 3–point interpolation formula for image texture enhancement [27]. The outcome was enhancement of both the image texture and lightness, and the information entropy was found to improve by 7%. Based on the Grünwald–Letnikov definition, Pu et al. derived some algorithms that perform well when applied to grey scale images, but produce distorted colour images [25]. They showed that their two algorithms YiFeiPU-1 and YiFeiPU-2 are widely applicable, particularly YiFeiPU-2, which using a Lagrange 3-point interpolation formula performs best based on a relative errors analysis. Based on this work, we extended the method to a second order Riesz fractional differential operator FCD-1 [8, 26]. The use of a fractional centered difference scheme enables our method to provide higher signal-to-noise ratios and superior image quality than the classical integral order differential mask operators and other first order fractional differential operators, such as YiFeiPU-1 [25]. These existing fractional order differentiation methods employ a fixed fractional order. Overall, fixed order techniques tend to excessively enhance the low spatial frequency content of an image, whilst insufficiently enhancing high spatial frequency content and amplifying image noise [3, 8, 24–26].

An optimization algorithm for choosing the fractional order parameter has been proposed to overcome limitations of fixed order methods [16]. Gilboa et al. adjusted the nonlinear diffusion coefficient locally according to image features, such as edges, textures and moments [28]. They illustrated that this approach works well, and sharpening and denoising can be combined together in the enhancement of grey–level and colour images. Huang et al. outlined an adaptive image enhancement algorithm that dynamically adjusts the fractional differential order according to the image local statistics and structural features [29]. Results were provided for the Lena image only, and a rigorous quantitative analysis of their findings was not performed. They did show that their method had a higher signal-to-noise ratio and superior image quality than the traditional fractional differential operator and classical integral order differential mask operators. In view of these findings, it appears that variable fractional order calculus provides benefits over fixed order methods. Hence, our work aims to further develop variable fractional order methods in the context of image enhancements.

In our previous work we showed that the use of the Riesz fractional differential operator instead of the Grünwald–Letnikov definition results in higher accuracy due to the positive benefits of using a symmetric second order instead of a one sided first order fractional operator [8, 26]. In view of these findings, we changed the YiFeiPU-2 method [25] by replacing the Grünwald–Letnikov definition with the Riesz fractional differential operator. Moreover, we incorporated variable fractional order [29] into the algorithm to estimate the fractal dimension of a local region instead of using fixed fractional order differentiation. The outcome is an image enhancement method applicable across a range of image types that adapts the fractional order of the differential to local features. As such, we achieve larger flexibility by being able to change the weights used in the reconstruction algorithm. The key differences of our method with previous work [8, 26] are that we apply a Lagrange 3-point interpolation formula on the Riesz fractional differential operator and incorporate variable fractional order into the algorithm. Key differences of the method with Pu et al. [25] are that we replace the Grünwald–Letnikov definition by the Riesz fractional differential operator and incorporate variable fractional order into the algorithm. Key differences of the method with Huang et al. [29] are that we replace the Grünwald–Letnikov definition by the Riesz fractional differential operator and apply a Lagrange 3-point interpolation formula on the Riesz fractional differential operator to achieve different effects based on the choice of the weights used in the reconstruction.

This paper is organized as follows. Firstly, the mathematical preliminaries used throughout the paper are introduced. Secondly, we give the theoretical analysis for our improved fractional differential mask, termed VOFCD, based on the second order Riesz fractional differential operator using a Lagrange 3-point interpolation formula. Then, the VOFCD method is presented. Furthermore, we present an overview of the data acquisition process and give an outline of the algorithm for enhancing the quality of a grey–level image. Finally, we compare our algorithm with Pu et al.’s best algorithm (YiFeiPU-2) [25] and Yu et al.’s algorithm (FCD-1) [8, 26] when applied to medical images of the human brain.

## Preliminary Knowledge

Here we outline the important definitions used throughout the paper.

**Definition 1**. The *v*-order Grünwald-Letnikov based fractional derivative ${}_{GL}D_{y}^{v}s(x,y)$ with respect to *x* for the finite interval *x* ∈ [*a*, *X*] can be expressed by [30, 31]:

$$\begin{array}{ccc} & & {}_{GL}D_{x}^{v}s\left(x,y\right)={\frac{d^{v}}{{\left[d(x-a)\right]}^{v}}s\left(x,y\right)|}_{GL}\\ & & =\lim_{N\to \infty }\{\frac{{\left(\frac{x-a}{N}\right)}^{-v}}{\Gamma (-v)}\sum_{k=0}^{N-1}\frac{\Gamma (k-v)}{\Gamma (k+1)}s(x-k(\frac{x-a}{N}))\}, \end{array}$$ (1)

where *v* is any real number. The *v*-order Grünwald-Letnikov based fractional derivative ${}_{GL}D_{y}^{v}s(x,y)$ with respect to *y* can be defined in a similar manner.

**Definition 2**. The *v*-order (0 < *v* ≤ 2) Riesz fractional derivative $\frac{\partial^{v}s(x,y)}{\partial {|x|}^{v}}$ with respect to *x* for the infinite interval −∞ < *x* < +∞ is defined as [8]:

$$\begin{array}{c} \frac{\partial^{v}s\left(x,y\right)}{\partial {\left|x\right|}^{v}}=-c_{v}(\frac{\partial^{v}}{\partial x^{v}}+\frac{\partial^{v}}{\partial {\left(-x\right)}^{v}})s\left(x,y\right), \end{array}$$ (2)

where c_*v*_ = [2 cos(*πv*/2)]^−1^ (*v* ≠ 1), *n* − 1 < *v* ≤ *n*, and

$$\begin{array}{ccc} \frac{\partial^{v}s\left(x,y\right)}{\partial x^{v}} & = & \frac{1}{\Gamma (n-v)}\frac{\partial^{n}}{\partial x^{n}}\int_{-\infty }^{x}\frac{s(\xi ,y)d\xi }{{\left(x-\xi \right)}^{v+1-n}}, \end{array}$$ (3)

$$\begin{array}{ccc} \frac{\partial^{v}s\left(t\right)}{\partial {\left(-x\right)}^{v}} & = & \frac{{\left(-1\right)}^{n}}{\Gamma (n-v)}\frac{\partial^{n}}{\partial x^{n}}\int_{x}^{+\infty }\frac{s(\xi ,y)d\xi }{{\left(\xi -x\right)}^{v+1-n}}. \end{array}$$ (4)

The Riesz fractional derivative $\frac{\partial^{v}s(x,y)}{\partial {|y|}^{v}}$ of order *v* (0 < *v* ≤ 2) with respect to *y* can be defined in a similar manner.

## The Fractional Differential Mask

Utilizing the second order fractional centered difference scheme [8, 26, 32], we discretize the Riesz fractional derivative ∂^*v*^ *s*(*x*, *y*)/∂∣*x*∣^*v*^ (0 < *v* ≤ 2) with respect to *x* with step *h* as:

$$\begin{array}{c} \frac{\partial^{v}s\left(x,y\right)}{\partial {\left|x\right|}^{v}}=-\frac{1}{h^{v}}\sum_{k=-\infty }^{\infty }\frac{{\left(-1\right)}^{k}\Gamma \left(v+1\right)}{\Gamma \left(\frac{v}{2}-k+1\right)\Gamma \left(\frac{v}{2}+k+1\right)}\cdot s\left(x-kh,y\right)+O\left(h^{2}\right). \end{array}$$ (5)

Assume that *r* = *x* + (*vh*/2) − *kh*, then *r* ∈ [*x* − *kh*, *x* + *h* − *kh*]. Using a Lagrange 3–point interpolation formula for the three neighboring nodes *s*(*x* + *h* − *kh*, *y*), *s*(*x* − *kh*, *y*), and *s*(*x* − *h* − *kh*, *y*), we have

$$\begin{array}{ccc} s(r,y) & ≅ & \frac{\left(r-x+kh)(r-x+h+kh\right)}{2h^{2}}s\left(x+h-kh,y\right)\\ & & -\frac{\left(r-x-h+kh)(r-x+h+kh\right)}{h^{2}}s\left(x-kh,y\right)\\ & & +\frac{\left(r-x-h+kh)(r-x+kh\right)}{2h^{2}}s\left(x-h-kh,y\right). \end{array}$$ (6)

Noting that *r* = *x* + (*vh*/2) − *kh*, we then obtain

$$\begin{array}{ccc} s(x+\frac{v}{2}h-kh,y) & ≅ & (\frac{v}{4}+\frac{v^{2}}{8})s\left(x+h-kh,y\right)+(1-\frac{v^{2}}{4})s\left(x-kh,y\right)\\ & & +(\frac{v^{2}}{8}-\frac{v}{4})s\left(x-h-kh,y\right). \end{array}$$ (7)

Compared with *s*(*x* − *kh*, *y*) in Eq (5), *s*(*x* + (*vh*/2) − *kh*, *y*) in Eq (7) is a linear combination of the neighboring nodes, which implies that *s*(*x* + (*vh*/2) − *kh*, *y*) contains more information in its neighborhood and leads to richer texture details. Thus, replacing *s*(*x* − *kh*, *y*) in Eq (5) with *s*(*x* + (*vh*/2) − *kh*, *y*), and substituting Eq (7) into Eq (5), we obtain a new Riesz fractional differential with respect to *x* as

$$\begin{array}{ccc} \frac{\partial^{v}s\left(x,y\right)}{\partial {\left|x\right|}^{v}} & ≅ & -\frac{1}{h^{v}}\sum_{k=-\infty }^{\infty }\frac{{\left(-1\right)}^{k}\Gamma \left(v+1\right)}{\Gamma \left(\frac{v}{2}-k+1\right)\Gamma \left(\frac{v}{2}+k+1\right)}\cdot s(x+\frac{v}{2}h-kh,y)\\ & ≅ & -\frac{1}{h^{v}}\sum_{k=-\infty }^{\infty }\frac{{\left(-1\right)}^{k}\Gamma \left(v+1\right)}{\Gamma \left(\frac{v}{2}-k+1\right)\Gamma \left(\frac{v}{2}+k+1\right)}\cdot [(\frac{v}{4}+\frac{v^{2}}{8})s\left(x+h-kh,y\right)\\ & & +(1-\frac{v^{2}}{4})s\left(x-kh,y\right)+(\frac{v^{2}}{8}-\frac{v}{4})s\left(x-h-kh,y\right)]. \end{array}$$ (8)

By first noting that for 0 < *v* < 1, $\Gamma (t)\Gamma (1-t)=\frac{\pi }{\text{sin}(\pi t)}$ (0 < *t* < 1), we have $\Gamma (\frac{v}{2})\Gamma (1-\frac{v}{2})=\frac{\pi }{\text{sin}(\frac{\pi v}{2})}$, and $\Gamma (v)\Gamma (1-v)=\frac{\pi }{\text{sin}(\pi v)}=\frac{\pi }{2\text{sin}(\frac{\pi v}{2})\text{cos}(\frac{\pi v}{2})}$ gives

$$\begin{array}{c} \frac{1}{2\cos(\frac{\pi v}{2})}=\frac{\Gamma (v)\Gamma (1-v)}{\Gamma \left(\frac{v}{2}\right)\Gamma \left(1-\frac{v}{2}\right)},\;0<v<1, \end{array}$$ (9)

we can rewrite Eq (8) in the form

$$\begin{array}{ccc} \frac{\partial^{v}s\left(x,y\right)}{\partial {\left|x\right|}^{v}} & = & -\frac{1}{2\cos(\frac{\pi v}{2})}(\frac{\partial^{v}}{\partial x^{v}}+\frac{\partial^{v}}{\partial {\left(-x\right)}^{v}})s\left(x,y\right)\\ & ≅ & -\frac{1}{2\cos\left(\frac{\pi v}{2}\right)h^{v}}\{\sum_{k=0}^{\infty }\omega_{k}[(\frac{v}{4}+\frac{v^{2}}{8})s\left(x+h-kh,y\right)+(1-\frac{v^{2}}{4})s\left(x-kh,y\right)\\ & & +(\frac{v^{2}}{8}-\frac{v}{4})s\left(x-h-kh,y\right)]+\sum_{k=-\infty }^{0}\omega_{k}[(\frac{v}{4}+\frac{v^{2}}{8})s\left(x+h-kh,y\right)\\ & & +(1-\frac{v^{2}}{4})s\left(x-kh,y\right)+(\frac{v^{2}}{8}-\frac{v}{4})s\left(x-h-kh,y\right)]\}, \end{array}$$ (10)

where

$$\begin{array}{ccc} \omega_{0} & = & -\frac{\Gamma (1-v/2)}{v\Gamma (1+v/2)\Gamma (-v)},\\ \omega_{k} & = & \frac{{\left(-1\right)}^{k+1}\Gamma \left(v/2\right)\Gamma \left(1-v/2\right)}{\Gamma (v/2-k+1)\Gamma (v/2+k+1)\Gamma (-v)}, \end{array}$$ (11)

for *k* = ±1, ±2, ⋯. It can be seen from Eq (10) that the Riesz fractional derivative can be discretized into two parts, one located on the positive *x*–axis and the other on the negative *x*–axis. From this formulation the Riesz fractional mask can be obtained.

In the context of medical images, the authors in [8, 25, 26] discuss the biggest variable of the grey level being limited, and the shortest distance for a change in the grey level image must be at an adjacent pixel. Therefore, the pixel signal is used to measure the duration of a two–dimensional digital image *s*(*x*, *y*) with respect to the two variables *x* and *y*. Here, the duration is the dimension of the image matrix assuming that the duration of *x* and *y* is [0, *X*] and [0, *Y*], respectively. The uniform distances for the *x* and *y*–coordinates are *h*_*x*_ = *X*/*N* = 1 and *h*_*y*_ = *Y*/*N* = 1 and the number of divisions are *N*_*x*_ = [*X*/*h*_*x*_] = [*X*] and *N*_*y*_ = [*Y*/*h*_*y*_] = [*Y*].

For a two–dimensional digital image *s*(*x*, *y*) at pixel signal (*x*_1_, *y*_1_) on the positive *x*–axis with region [0, *x*_1_ + *h*], the *N* + 2 pixels are

$$\begin{array}{ccc} & & s_{N}\left(x_{1},y_{1}\right)=s\left(0,y_{1}\right),\;\cdots ,\;s_{k}\left(x_{1},y_{1}\right)=s\left(x_{1}-kh,y_{1}\right),\;\cdots ,\\ & & s_{0}\left(x_{1},y_{1}\right)=s\left(x_{1},y_{1}\right),\;s_{-1}\left(x_{1},y_{1}\right)=s\left(x_{1}+h,y_{1}\right). \end{array}$$

After truncation, the anterior *n* + 2 approximate fractional centered difference of the Riesz fractional differential with order 0 < *v* < 1on the positive *x*–axis is:

$$\begin{array}{ccc} \frac{\partial^{v}s\left(x,y\right)}{\partial x^{v}} & ≅ & -\frac{1}{2\cos\left(\frac{\pi v}{2}\right)h^{v}}\sum_{k=0}^{n-1}\omega_{k}[(\frac{v}{4}+\frac{v^{2}}{8})s\left(x+h-kh,y\right)\\ & & +(1-\frac{v^{2}}{4})s\left(x-kh,y\right)+(\frac{v^{2}}{8}-\frac{v}{4})s\left(x-h-kh,y\right)]. \end{array}$$ (12)

Similarly, the anterior *n* + 2 approximate fractional centered difference of the Riesz fractional differential with order 0 < *v* < 1on the negative *x*–axis is:

$$\begin{array}{ccc} \frac{\partial^{v}s\left(x,y\right)}{\partial x^{v}} & ≅ & -\frac{1}{2\cos\left(\frac{\pi v}{2}\right)h^{v}}\sum_{k=-(n-1)}^{0}\omega_{k}[(\frac{v}{4}+\frac{v^{2}}{8})s\left(x+h-kh,y\right)\\ & & +(1-\frac{v^{2}}{4})s\left(x-kh,y\right)+(\frac{v^{2}}{8}-\frac{v}{4})s\left(x-h-kh,y\right)]. \end{array}$$ (13)

**Fig 1:**
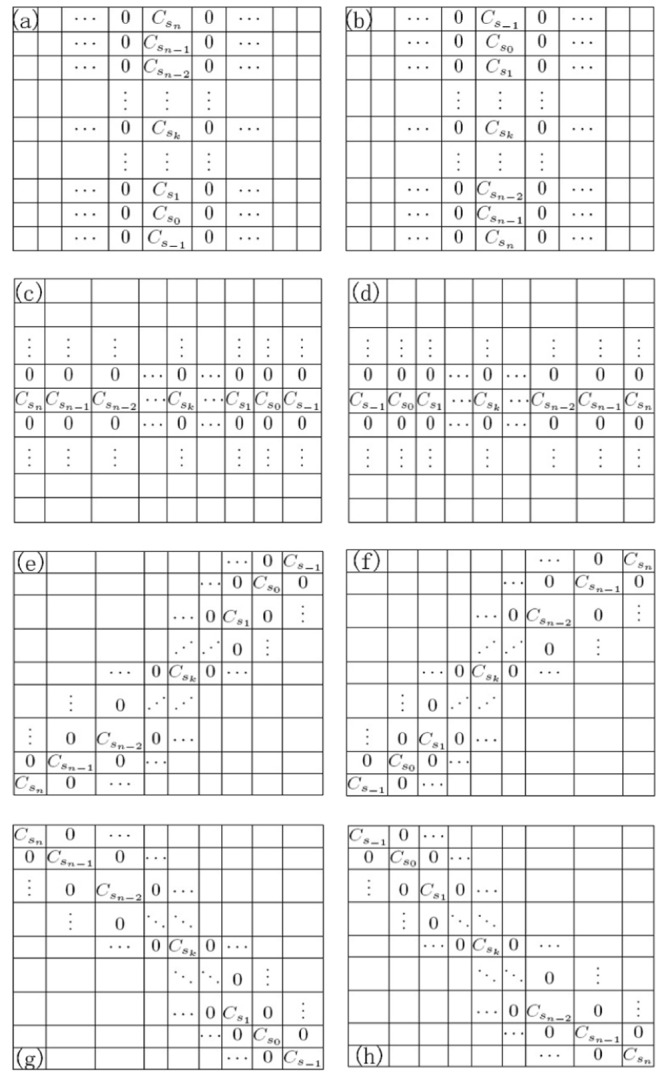
Fractional differential operator VOFCD for the eight directions. (a) *W*_1_(negative *x*–axis), (b) *W*_2_(positive *x*–axis), (c) *W*_3_(negative *y*–axis), (d) *W*_4_ (positive *y*–axis), (e) *W*_5_(left downward diagonal), (f) *W*_6_ (right upward diagonal), (g) *W*_7_(left upward diagonal), (h) *W*_8_ (right downward diagonal).

To obtain the fractional differential masks for the eight symmetric directions and make them rotationally invariant, we implement eight fractional differential masks positioned respectively on the negative *x*–axis, positive *x*–axis, negative *y*–axis, positive *y*–axis, left downward diagonal, right upward diagonal, left upward diagonal, and right downward diagonal. They are correspondingly denoted by *W*_*l*_ (*l* = 1, 2, …, 8) (see Fig 1).

In Fig 1, *C*_*s*_0__ is the mask coefficient associated with the pixel of interest. When *n* = 2*m* − 1, a(2*m* + 1) × (2*m* + 1) fractional differential mask is implemented. To ensure that the fractional differential mask remains symmetric and the centre of the mask aligns with a pixel, in general, *n* is taken as an odd positive integer.

Digital image processing is based on direct processing for discrete pixels, and the algorithm normally adopts an airspace filtering scheme whose principle is to move the mask pixel by pixel [25]. Therefore, there are separate algorithms for grey and colour image fractional differential masks.

Next, we deduce the following fractional differential algorithm, VOFCD, based on the Riesz fractional differential operator Eq (2). To treat the *N*_*x*_ × *N*_*y*_ digital grey–level image *s*(*x*, *y*), we perform a convolution filter on the above eight directions in the (2*m* + 1) × (2*m* + 1) masks, and propose that the eight fractional differential masks are computed by what we refer to as the VOFCD operator:

$$\begin{array}{ccc} s_{l_{1}}\left(x,y\right) & = & \sum_{i=M_{l_{1}}}^{N_{l_{1}}}\sum_{j=P_{l_{1}}}^{Q_{l_{1}}}W_{l_{1}}\left(i,j\right)s\left(x+i,y+j\right), \end{array}$$ (14)

$$\begin{array}{ccc} s_{l_{2}}\left(x,y\right) & = & \sum_{i=M_{l_{2}}}^{N_{l_{2}}}\sum_{j=P_{l_{2}}}^{Q_{l_{2}}}2^{-v/2}W_{l_{2}}\left(i,j\right)s\left(x+i,y+j\right)\\ & & +\left(1-2^{-v/2}\right)W_{l_{2}}\left(0,0\right)s\left(x,y\right), \end{array}$$ (15)

where *l*_1_ = 1, 2, 3, 4, *l*_2_ = 5, 6, 7, 8, and

$$\begin{array}{cccccccc} & M_{1}=-2m, & & N_{1}=1, & & P_{1}=-m, & & Q_{1}=m;\\ & M_{2}=-1, & & N_{2}=2m, & & P_{2}=-m, & & Q_{2}=m;\\ & M_{3}=-m, & & N_{3}=m, & & P_{3}=-2m, & & Q_{3}=1;\\ & M_{4}=-m, & & N_{4}=m, & & P_{4}=-1, & & Q_{4}=2m;\\ & M_{5}=-1, & & N_{5}=2m, & & P_{5}=-2m, & & Q_{5}=1;\\ & M_{6}=-2m, & & N_{6}=1, & & P_{6}=-1, & & Q_{6}=2m;\\ & M_{7}=-2m, & & N_{7}=1, & & P_{7}=-2m, & & Q_{7}=1;\\ & M_{8}=-1, & & N_{8}=2m, & & P_{8}=-1, & & Q_{8}=2m. \end{array}$$

Thus, we have the digital grey–level images *s*_*I*_(*x*, *y*)as

$$\begin{array}{c} s_{I}\left(x,y\right)=\frac{\sum_{l=1}^{8}s_{l}\left(x,y\right)}{4\sum_{k=-1}^{n}C_{s_{k}}+\sum_{k=-1,k\neq 0}^{n}2^{(4-v)/2}C_{s_{k}}+4C_{s_{0}}}, \end{array}$$ (16)

where *C*_*s*_*k*__ is the mask coefficient given in Eq (17). As for the digital colour image, the algorithm is similar to that for a grey–level image, however the RGB components use the fractional differential respectively.

When 0 < *v* < 1, we implement the fractional differential mask respectively on the eight symmetric directions using what we call the VOFCD operator, having the same structure as YiFeiPU-2 in [25] but with different coefficients. The mask coefficients of the VOFCD operator are given by

$$\begin{array}{ccc} C_{s_{-1}} & = & -\frac{1}{2\cos\left(\pi v/2\right)h^{v}}(\frac{v}{4}+\frac{v^{2}}{8})\omega_{0},\\ C_{s_{0}} & = & -\frac{1}{2\cos\left(\pi v/2\right)h^{v}}[(1-\frac{v^{2}}{4})\omega_{0}+(\frac{v}{4}+\frac{v^{2}}{8})\omega_{1}],\\ \vdots \\ C_{s_{k}} & = & -\frac{1}{2\cos\left(\pi v/2\right)h^{v}}[(\frac{v^{2}}{8}-\frac{v}{4})\omega_{k-1}+(1-\frac{v^{2}}{4})\omega_{k}+(\frac{v}{4}+\frac{v^{2}}{8})\omega_{k+1}],\\ \vdots \\ C_{s_{n-1}} & = & -\frac{1}{2\cos\left(\pi v/2\right)h^{v}}[(\frac{v^{2}}{8}-\frac{v}{4})\omega_{n-2}+(1-\frac{v^{2}}{4})\omega_{n-1}],\\ C_{s_{n}} & = & -\frac{1}{2\cos\left(\pi v/2\right)h^{v}}(\frac{v^{2}}{8}-\frac{v}{4})\omega_{n-1}, \end{array}$$ (17)

which ensures that the fractional differential operator VOFCD produces a sparse matrix having dimension *n* + 2. Moreover, all the coefficients depend on the fractional differential order *v*. It can also be proven that the sum of the coefficients is nonzero, which is the main difference between the fractional differential mask and the integral version.

## Variable Fractional Differential Order

The average gradient and information entropy are widely used for quantitative analysis of images [23, 25, 27]. The average gradient reflects the clarity of the image, and expresses contrast due to small details. It can be used to measure the spatial resolution of the image, i.e., a larger average gradient means a higher spatial resolution [33–35]. Generally speaking, a large gradient value is likely to correspond to regions of edges, margins and textures within images, hence a larger fractional order is required to enhance textural details [8, 25, 26]. On the contrary, a smaller value of the average gradient is likely to correspond to smooth image regions, hence a smaller fractional order is needed to enhance image textures. However, the gradient is quite large for both boundaries and noise. Therefore, a suitable method is needed to identify the margin and noise.

Note that the margin of the object is continuously smooth, and it is easy to find another margin point that has a similar magnitude of gradient as the margin of interest. However, the noise is random, and it is difficult to find any noise around the region of interest having a similar magnitude of gradient. Hence, margin or noise can be identified through the following process:

$$\begin{array}{c} R=\{\begin{array}{c} 0,\;\min\{∥\nabla s_{ij}-\nabla N\left(s_{ij}\right)∥\}\leq T\\ 1,\;\min\{∥\nabla s_{ij}-\nabla N\left(s_{ij}\right)∥\}>T \end{array} \end{array}$$ (18)

where ∇*s*_*ij*_ is the gradient magnitude associated with the pixel of interest *s*(*x*_*i*_, *y*_*j*_), ∇*N*(*s*_*ij*_) is the gradient magnitude associated with the pixel around the region of interest *s*(*x*_*i*_, *y*_*j*_), and *T* is the threshold value.

The information entropy evaluates the average information included in the image and reflects the detail information of the image [23, 25, 27, 36–38], that is small in the smooth area and large in the rich texture area of the image. Image entropy is calculated as:

$$\begin{array}{c} H=-\sum_{i,j}P\left(x_{i},y_{j}\right)\log_{2}P\left(x_{i},y_{j}\right), \end{array}$$ (19)

where *P*(*x*_*i*_, *y*_*j*_) is the probability mass function of *s*(*x*_*i*_, *y*_*j*_).

The local image roughness is the relative offset measurement of the image pixel grey scale value [36, 39, 40], that is also small in the smooth area and large in the rich texture area of the image. The local roughness of an image is calculated as

$$\begin{array}{c} Q=1-\frac{1}{1+\sigma^{2}}, \end{array}$$ (20)

where *σ* is the local variance of the pixel signals of the image.

The order of the fractional differential is not only related to the magnitude of the gradient, but also impacted by the image local statistics, namely variance and entropy [23, 25, 27]. Thus, using a weighted summation of three parameters to reflect local image information, we have:

$$\begin{array}{c} g\left(|\nabla s|,H,Q\right)=k_{1}∥\nabla s∥+k_{2}H+k_{3}Q, \end{array}$$ (21)

where 0 ≤ *k*_1_, *k*_2_, *k*_3_ ≤ 1 and *k*_1_ + *k*_2_ + *k*_3_ = 1.

As suggested by Huang et al. [29], utilizing the exponential function property, we can obtain the variable fractional differential order function as:

$$\begin{array}{c} v={\left(-1\right)}^{R}(e^{\alpha g(∥\nabla s∥,H,Q)}-\beta ), \end{array}$$ (22)

where *α* and *β* are regularization parameters.

## Data Acquisition and Algorithm Development

In this section, we outline the implementation of our algorithm to enhance textures in grey level and colour images. We first present the details of the data acquisition. Then, we demonstrate the algorithm development.

### Data Acquisition

The research project was approved by the Human Research Ethics Committee of Queensland Health, Brisbane, Australia. Participants provided written consent prior to participating in the study, the process of which was approved by the ethics committee. Furthermore, all data sets were de-identified for the project.

**Fig 2:**
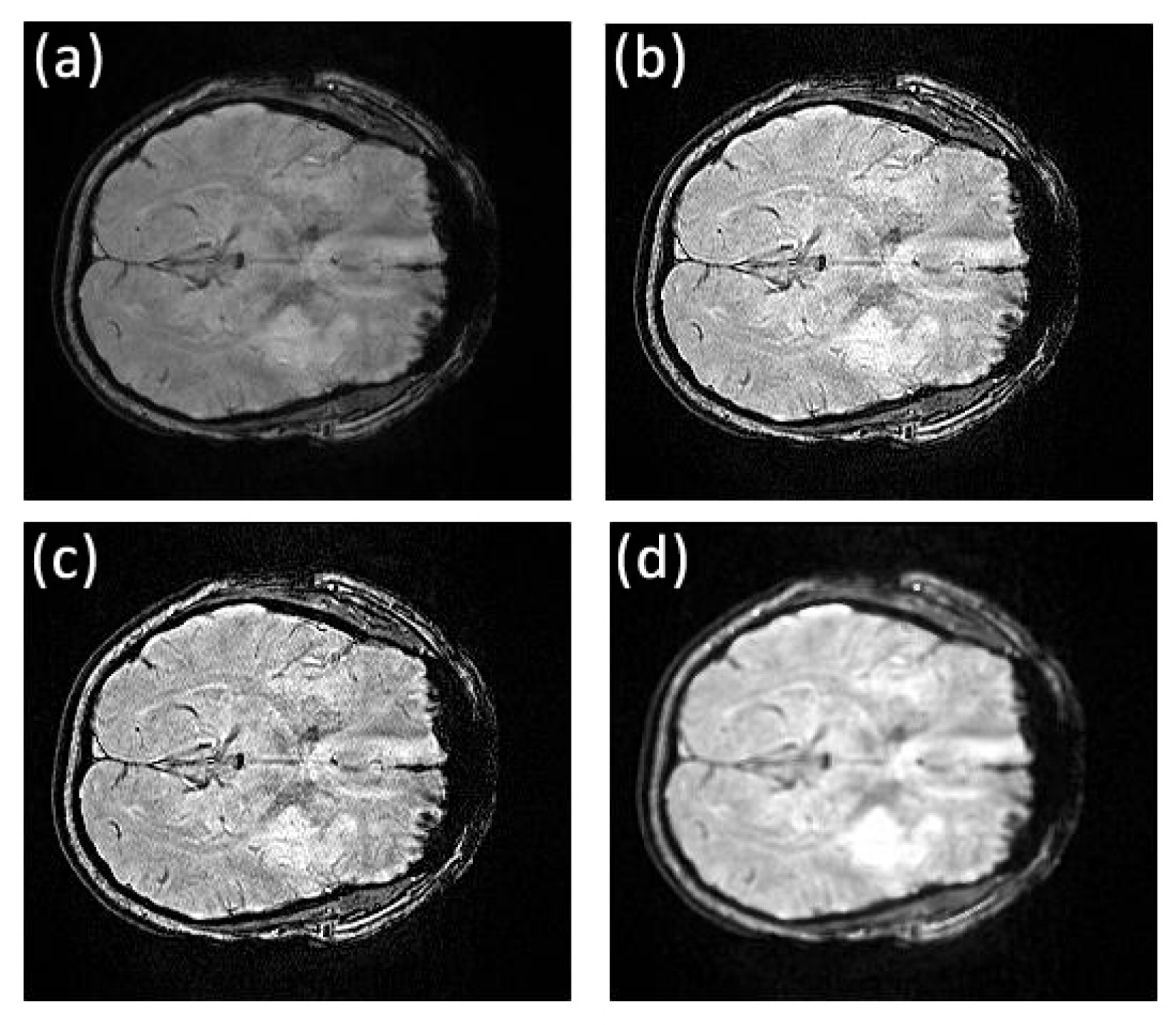
Comparison of texture details between original image slice 35 from the first stroke patient and its fractional differential using YiFeiPU-2, FCD-1 and VOFCD and (0.45, 0.01, 0.54) for the weights. (a) Original image slice 35, (b) 0.5–order YiFeiPU-2 with mask 5 × 5, (c) 0.5–order FCD-1 with mask 5 × 5, (d) VOFCD with mask 5 × 5.

**Fig 3:**
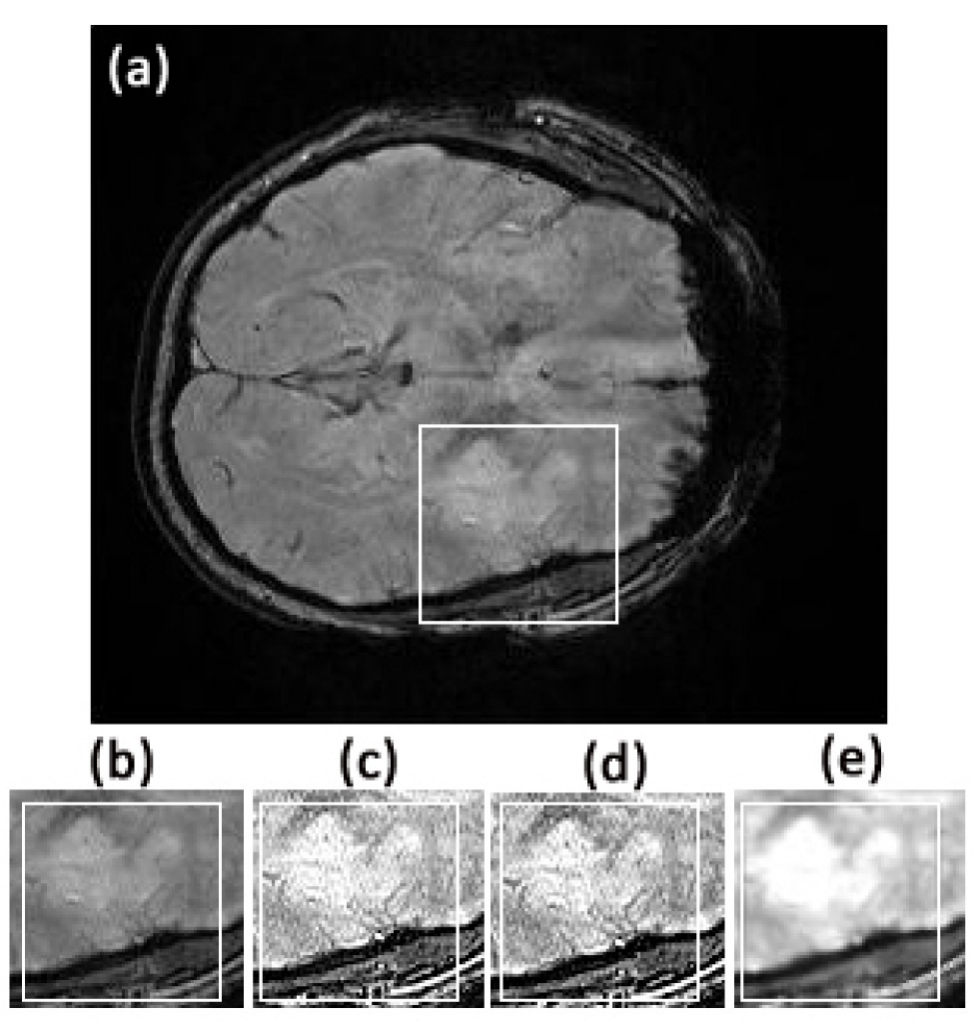
Comparison of textures in the region of interest between original image slice 35 from the first stroke patient and its fractional differential using YiFeiPU-2, FCD-1 and VOFCD and (0.45, 0.01, 0.54) for the weights. (a) Original full image slice 35, (b) original region of interest, (c) 0.5–order YiFeiPU-2 with mask 5 × 5, (d)0.5–order FCD-1 with mask 5 × 5, (e) VOFCD with mask 5 × 5.

The first data set of the stroke patients was acquired at the Royal Brisbane and Women’s Hospital using the 3T Siemens MRI scanner. Patients admitted with a clinical diagnosis of acute ischemic stroke were recruited between May 2011 and April 2012. Patients underwent an MRI examination at admission from which two MRI datasets were randomly selected for this study. Susceptibility weighted magnetic resonance images were acquired on a 3T Siemens Trio human scanner running the Syngo proprietary software housed at the hospital. Data acquisition was performed using the Syngo SWI sequence with the following parameters: matrix size = 224 × 256, repetition time (TR) = 200ms, echo time (TE) = 20ms, flip angle = 15^*o*^, bandwidth = 120Hz per pixel, in-plane resolution, 1mm × 1mm slice thickness and separation = 2mm and number of slices = 72. Magnitude, phase and susceptibility images were saved separately, and only the magnitude images were used in this study. The original image slices 32 to 47 from the first stroke patient show the stroke in the left hemisphere of the brain, as for example, can be seen in Figs 2(a) and 3(a). For the second patient, the stroke was visible in slices 42 to 61 to various extents.

The second data set is for MRI data acquisition–3T in vivo for a patient with Parkinson’s disease. The data was obtained from St Andrew’s War Memorial Hospital, Brisbane, Australia. The pre-surgery data we use here is from a patient diagnosed with Parkinson’s disease. The brain diffusion tensor MR images are acquired using a GE Medical System (SIGNA 3T) scanner. All image matrix sizes are 256 × 256. Pre surgery data is acquired using an echo time of 93.7 ms and repetition time of 7s. Twenty four interleaved, 5 mm thick slices are acquired in the horizontal plane perpendicular to the coronal and sagittal planes, using the multislice mode. Diffusion sensitization is performed along 35 different diffusion gradient orientations using a diffusion weighting of *b* = 1000 sec/mm^2^ (*b* is the degree of diffusion sensitization defined by the amplitude and the time course of the magnetic field gradient pulses used to encode molecular diffusion displacements [41]). A reference image without diffusion weighting (*b* = 0 sec/mm^2^) not illustrated here was also acquired.

### Image Evaluation

The regulation parameters are set to *α* = 1 and *β* = 1.7, which gives the variable fractional differential order function as:

$$\begin{array}{c} v={\left(-1\right)}^{R}(e^{k_{1}∥\nabla s∥+k_{2}H+k_{3}Q}-1.7). \end{array}$$ (23)

After normalization with the magnitude of the gradient ‖∇*s*‖, the entropy of the image *H* and the roughness of the image *Q*, we have 0 ≤ ‖∇*s*‖, *H*, *Q* ≤ 1. Hence, if *R* = 0, we have *v* ∈ [0, *e* − 1.7] which effectively enhance textures in images, where *e* is the base of the natural logarithm and approximately equal to 2.71828; If *R* = 1, we have *v* ∈ [1.7 − *e*, 0] which can remove noise to some extent.

We define the signal to noise ratio (SNR) [26]

$$\begin{array}{c} \text{SNR}={\left(A_{signal}/A_{noise}\right)}^{2}, \end{array}$$ (24)

where *A* is the root mean square amplitude. For MRI data, the image background was used to compute *A*_*noise*_.

Images are evaluated based on the intensity histogram Entropy (Ent), Standard Deviation (STD), and Mean Absolute Difference Coefficient (MADC) [42]. The higher the value of Ent, the more visual information the image contains, and an increase in Ent means an increase in information and therefore an improvement in image quality. The STD demonstrates how much the image intensities deviate from the expected value, and a variation of STD measures how much corresponding points vary across the source and images. The MADC is used to measure image clarity, such as activity, and is defined as the mean of the sum of difference values over rows and columns of the image pixel intensities [42, 43]:

$$\begin{array}{c} \text{MADC}=\sqrt{\sum_{x}\sum_{y}(I_{x}^{2}+I_{y}^{2})/\left(N_{x}N_{y}\right)}, \end{array}$$ (25)

where *I*_*x*_ = [*s*(*x*, *y*) − *s*(*x* − 1, *y*)]^2^ and *I*_*y*_ = [*s*(*x*, *y*) − *s*(*x*, *y* − 1)]^2^.

### Simulation Study

The algorithm given in Table 1 provides the process for enhancing the quality of a grey-level image using the symmetric Riesz variable fractional order substitution.

**Table 1:** Algorithm for enhancing the quality of a grey–level image.

**Input**: Read original grey image, and add Gaussian noise with mean 0 and variance 0.01.
**Output**: *s*(*x*, *y*)
Choose *m* = 2, *n* = 3 and mask 5 × 5 Compute the variable fractional differential order using Eq (23) Compute the mask coefficients **C**_**s**_**k**__ using Eq (17) Compute the dimension *N*_*x*_ × *N*_*y*_ of the image matrix **for** *x* = 2:*N*_*x*_ − 1 **do**
**for** *y* = 2:*N*_*y*_ − 1 **do**
**for** *l* = 1:8 **do**
Compute *s*_*l*_(*x*, *y*) using Eqs (14) and (15)
**end**
Compute *s*_*I*_(*x*, *y*) using Eq (16)
**end**
**end**
Display adjusted image *s*_*I*_(*x*, *y*).

## Results and Discussion

In this section, we compare our algorithm to YiFeiPU-2 [25] and FCD-1 [8, 26], and apply the method to a set of medical images.

### Evaluation of Weights

**Fig 4:**
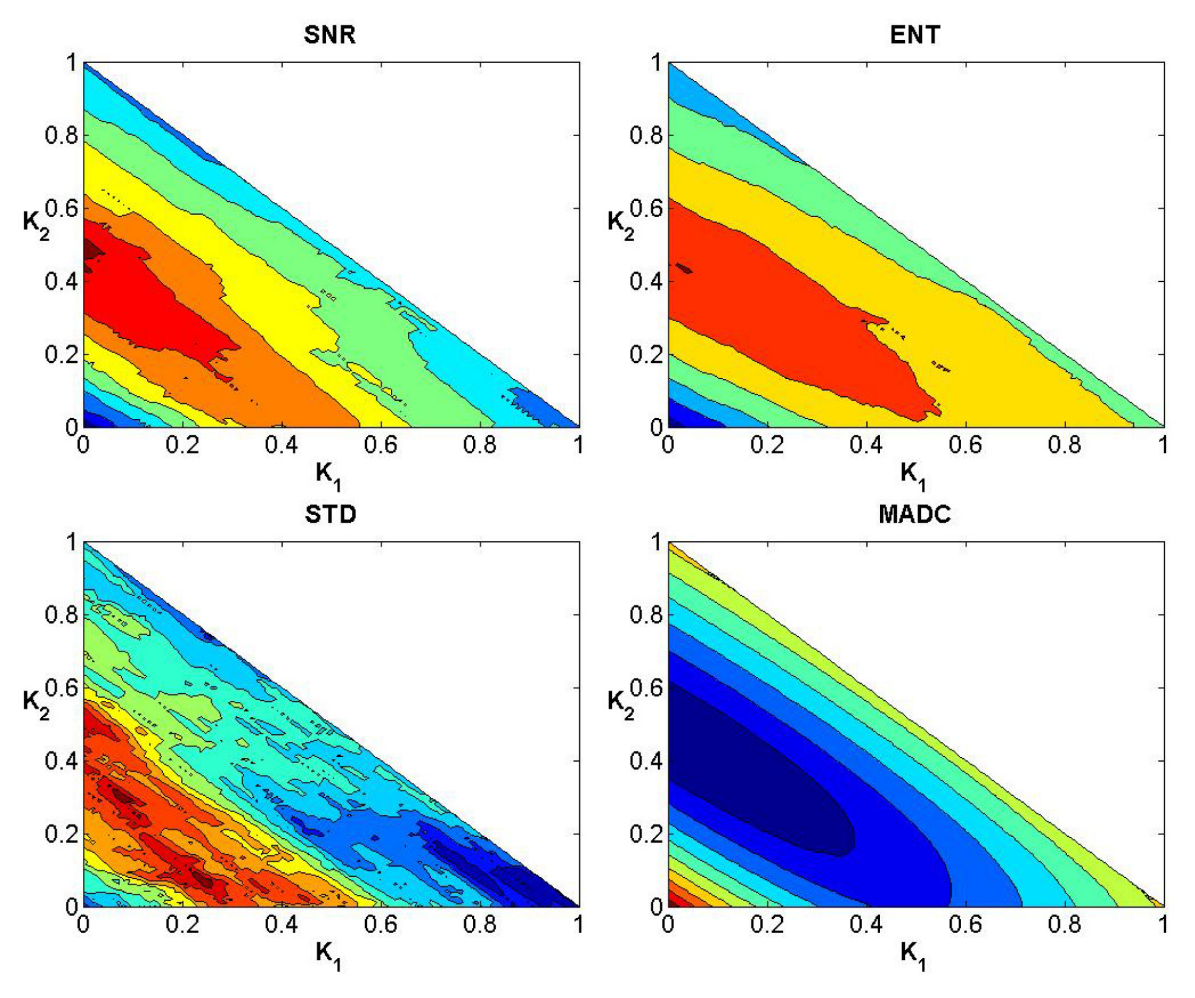
Convergence curves for SNR, Ent, STD and MADC using a 3 × 3 mask for image slice 35 for the first stroke patient.

**Fig 5:**
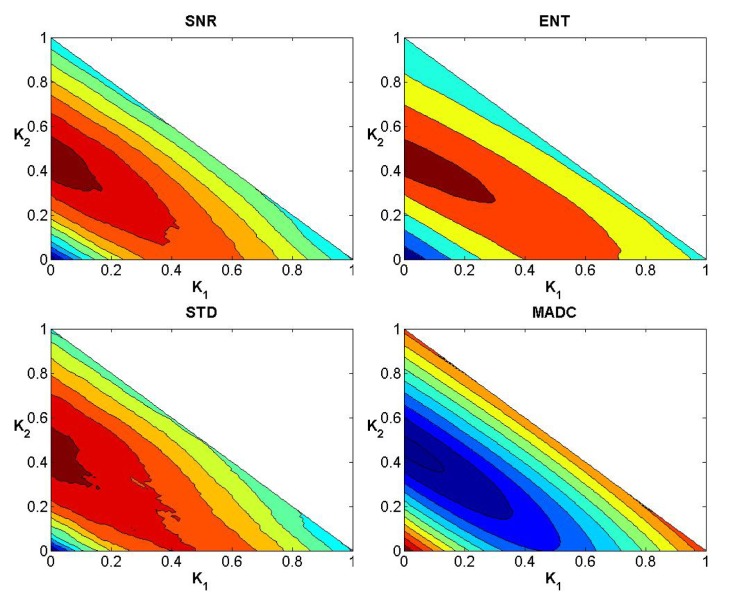
Convergence curves for SNR, Ent, STD and MADC using a 5 × 5 mask for image slice 35 for the first stroke patient.

**Fig 6:**
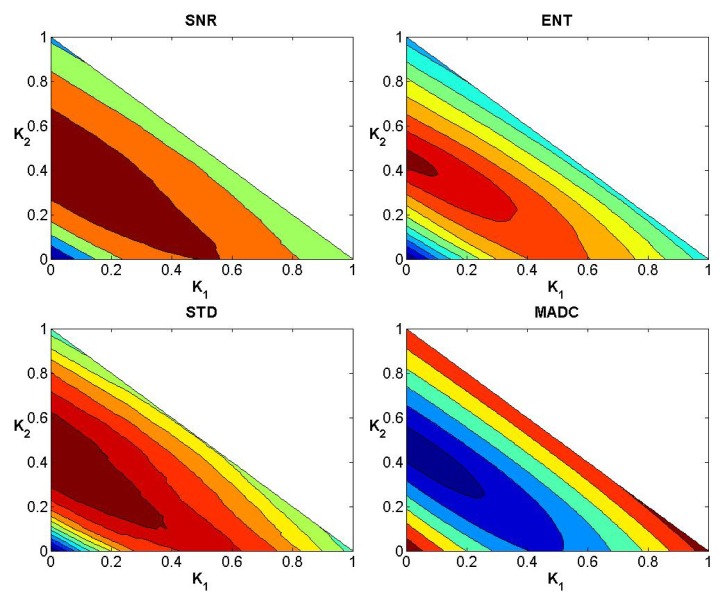
Convergence curves for SNR, Ent, STD and MADC using a 7 × 7 mask for image slice 35 for the first stroke patient.

After testing weights *k*_1_, *k*_2_ and *k*_3_ ranging from 0 to 1 with step 0.01 subject to the condition *k*_1_ + *k*_2_ + *k*_3_ = 1, Figs 4–6 show the convergence curves of SNR, Ent, STD and MADC with different mask sizes in image slice 35 for the first stroke patient (see Fig 2(a)). Using a 5 × 5 mask, Tables 2 and 3 show different combinations of *k*_1_, *k*_2_ and *k*_3_ for obtaining the largest and lowest SNR, Ent, STD and MADC.

**Table 2:** Weights *k*_1_, *k*_2_ and *k*_3_ to obtain the largest SNR, Ent, STD and MADC using various mask sizes. Data taken from image slice 35 for the first stroke patient.

	**Mask = 3 × 3**				**Mask = 5 × 5**				**Mask = 7 × 7**			
	Value	*k*_1_	*k*_2_	*k*_3_	Value	*k*_1_	*k*_2_	*k*_3_	Value	*k*_1_	*k*_2_	*k*_3_
SNR	105.1185	0.47	0.01	0.52	104.5495	0.45	0.01	0.54	103.9047	0.47	0.01	0.52
Ent	6.5951	0.42	0.05	0.53	6.6704	0.45	0.01	0.54	6.6626	0.45	0.01	0.54
STD	0.3563	0.07	0.25	0.68	0.3552	0.5	0.01	0.49	0.3547	0.49	0.01	0.5
MADC	0.06785	0.01	0.01	0.98	0.1247	0.01	0.01	0.98	0.1385	0.01	0.01	0.98

**Table 3:** Weights *k*_1_, *k*_2_ and *k*_3_ to obtain the lowest SNR, Ent, STD and MADC using various mask sizes. Data taken from image slice 35 for the first stroke patient.

	**Mask = 3 × 3**				**Mask = 5 × 5**				**Mask = 7 × 7**			
	Value	*k*_1_	*k*_2_	*k*_3_	Value	*k*_1_	*k*_2_	*k*_3_	Value	*k*_1_	*k*_2_	*k*_3_
SNR	104.3209	0.01	0.01	0.98	94.2579	0.01	0.01	0.98	79.2511	0.01	0.01	0.98
Ent	6.5666	0.01	0.01	0.98	6.3978	0.01	0.01	0.98	6.1545	0.01	0.01	0.98
STD	0.3552	0.01	0.98	0.01	0.3352	0.01	0.01	0.98	0.2958	0.01	0.01	0.98
MADC	0.05804	0.45	0.03	0.52	0.068907	0.46	0.01	0.53	0.07590	0.44	0.04	0.52

**Fig 7:**
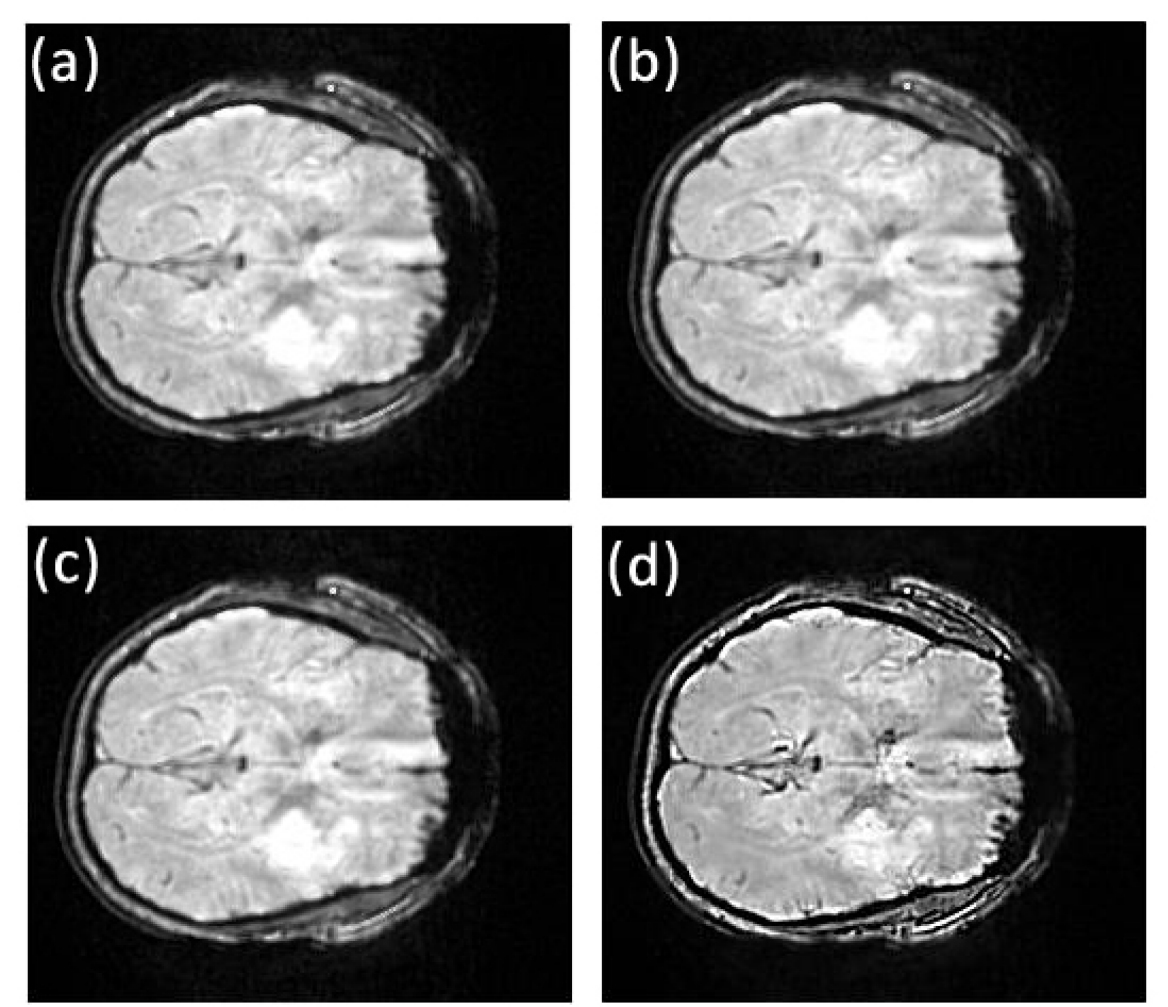
Comparison of images across different combinations of (*k*_1_, *k*_2_, *k*_3_) reconstructed using a 5 × 5 mask. Data is for slice 35 from the first stroke patient, and the weights are (a) (0.45, 0.01, 0.54), (b) (0.46, 0.01, 0.53), (c) (0.5, 0.01, 0.49), (d) (0.01, 0.01, 0.98).

According to the different combinations of *k*_1_, *k*_2_ and *k*_3_ from Tables 2 and 3, Fig 7 shows the corresponding image slice.

**Fig 8:**
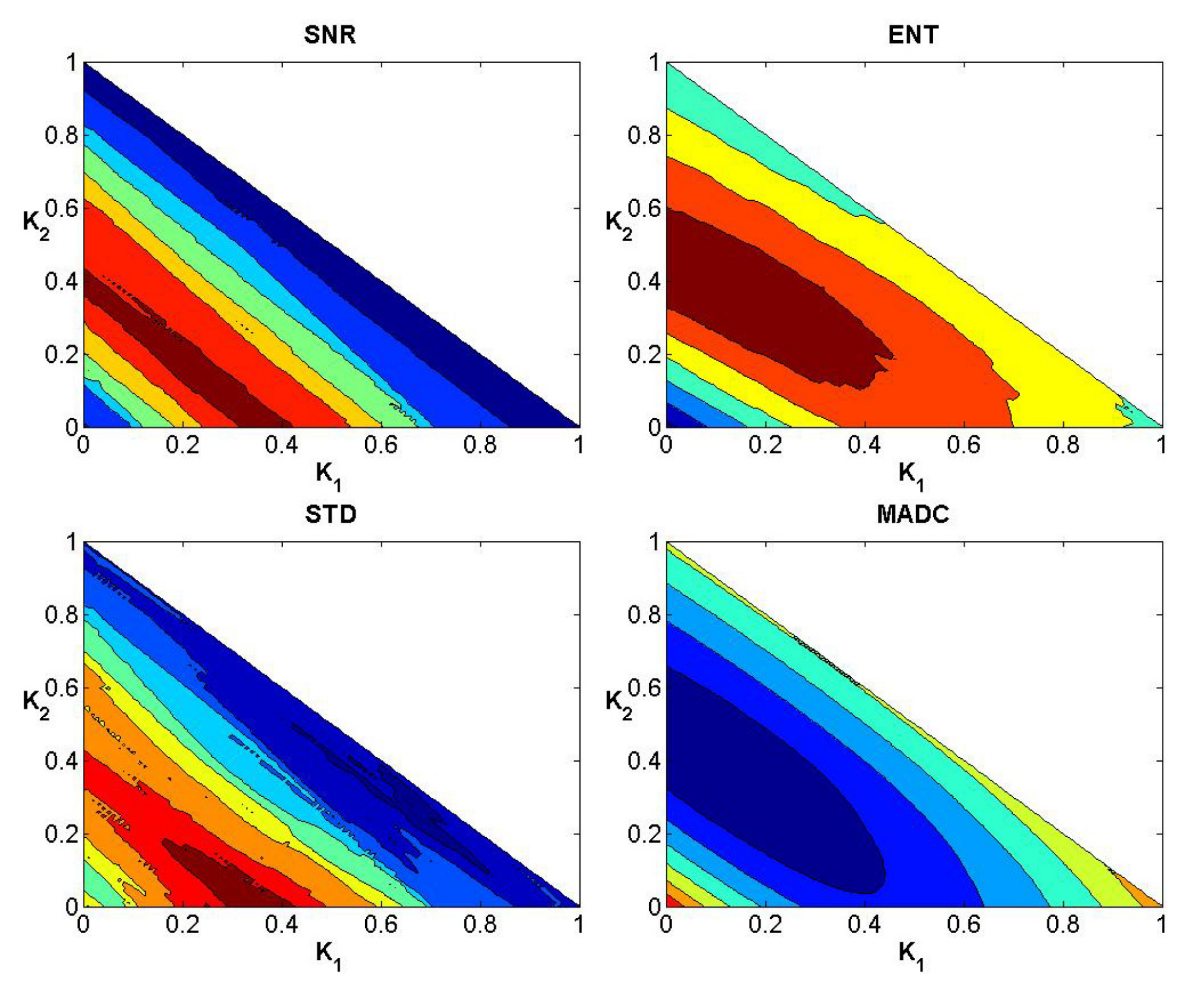
Convergence curves for SNR, Ent, STD and MADC using a 3 × 3 mask for image slice 51 from the second stroke patient.

**Fig 9:**
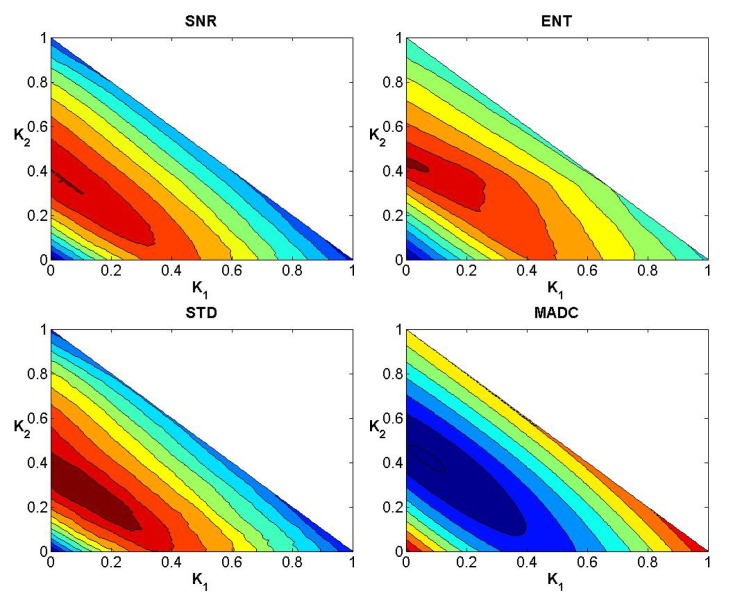
Convergence curves for SNR, Ent, STD and MADC using a 5 × 5 mask for image slice 51 from the second stroke patient.

**Fig 10:**
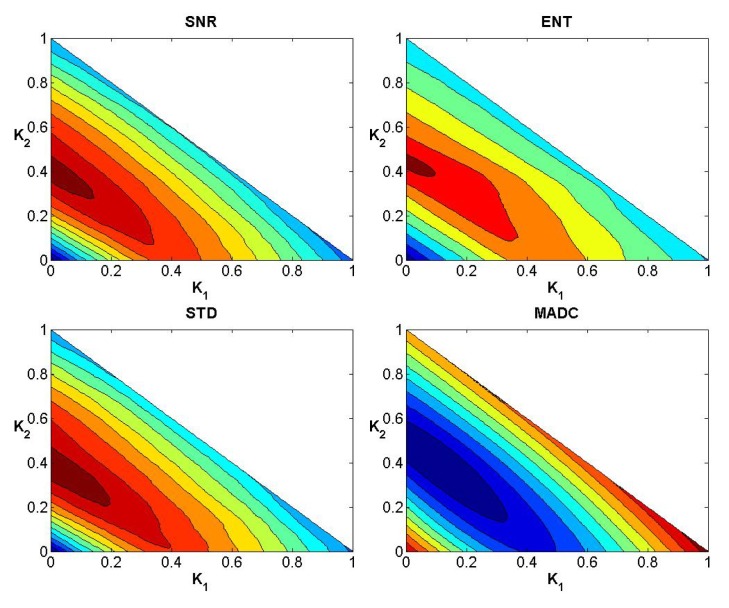
Convergence curves for SNR, Ent, STD and MADC using a 7 × 7 mask for image slice 51 from the second stroke patient.

**Fig 11:**
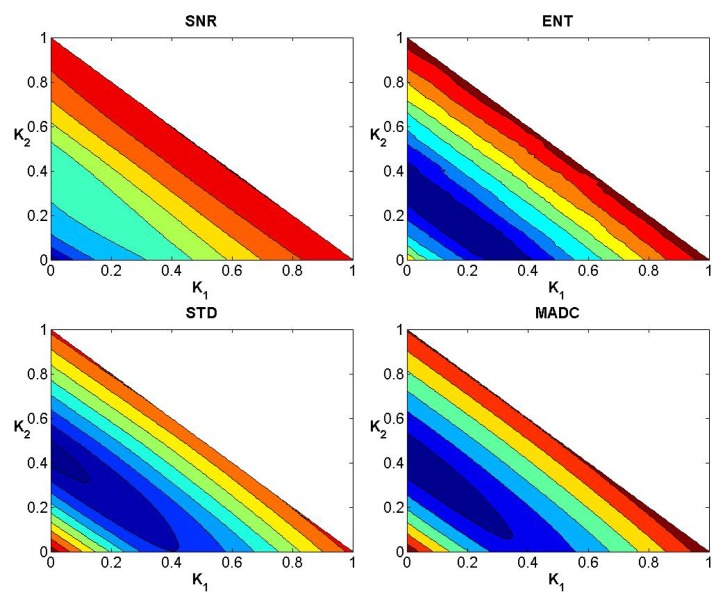
Convergence curves for SNR, Ent, STD and MADC using a 3 × 3 mask for an original fractional anisotropy weighted orientation map from a patient diagnosed with Parkinson’s disease prior to surgery.

**Fig 12:**
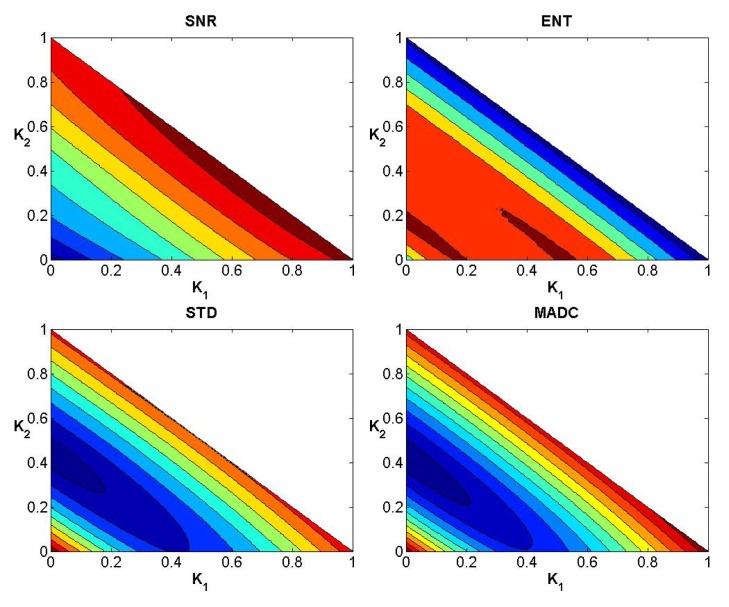
Convergence curves for SNR, Ent, STD and MADC using a 5 × 5 mask for an original fractional anisotropy weighted orientation map from a patient diagnosed with Parkinson’s disease prior to surgery.

**Fig 13:**
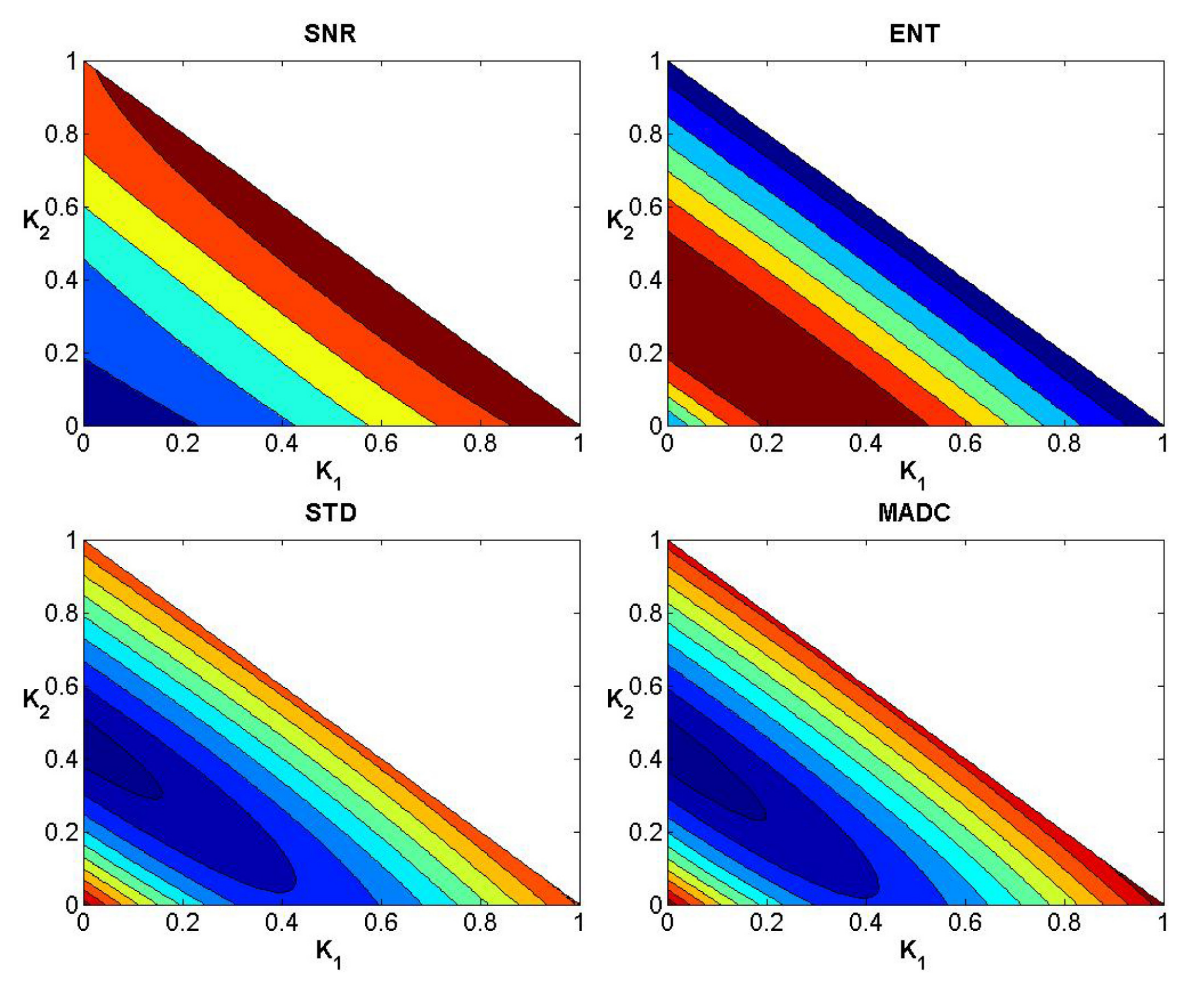
Convergence curves for SNR, Ent, STD and MADC using a 7 × 7 mask for an original fractional anisotropy weighted orientation map from a patient diagnosed with Parkinson’s disease prior to surgery.

In a similar manner to the data exhibited for the first stroke patient, Figs 8–10 show the convergence curves for SNR, Ent, STD and MADC with different mask sizes for image slice 51 from the second stroke patient. Figs 11–13 show the corresponding results from clinical data for a patient diagnosed with Parkinson’s disease prior to surgery.

Additional experiments were performed on different image slices of stroke patients, the results of which are provided as Supporting Information.

We have shown that the outlined approach can be applied to different data, and the weights can be manipulated to achieve the highest or lowest SNR, Ent, STD or MADC.

### Qualitative Analysis

Without loss of generality, we only show results using the 5 × 5 mask applied to the first stroke patient image using two particular combinations of *k*_1_, *k*_2_ and *k*_3_. The results of the second stroke patient are provided as Supporting Information. The parameter set (0.45, 0.01, 0.54) obtained the largest SNR in slice 35 depicted in Fig 7, whilst (0.01, 0.01, 0.98) produced the largest MADC. These two choices of parameters allow us to evaluate image SNR versus contrast.

**Fig 14:**
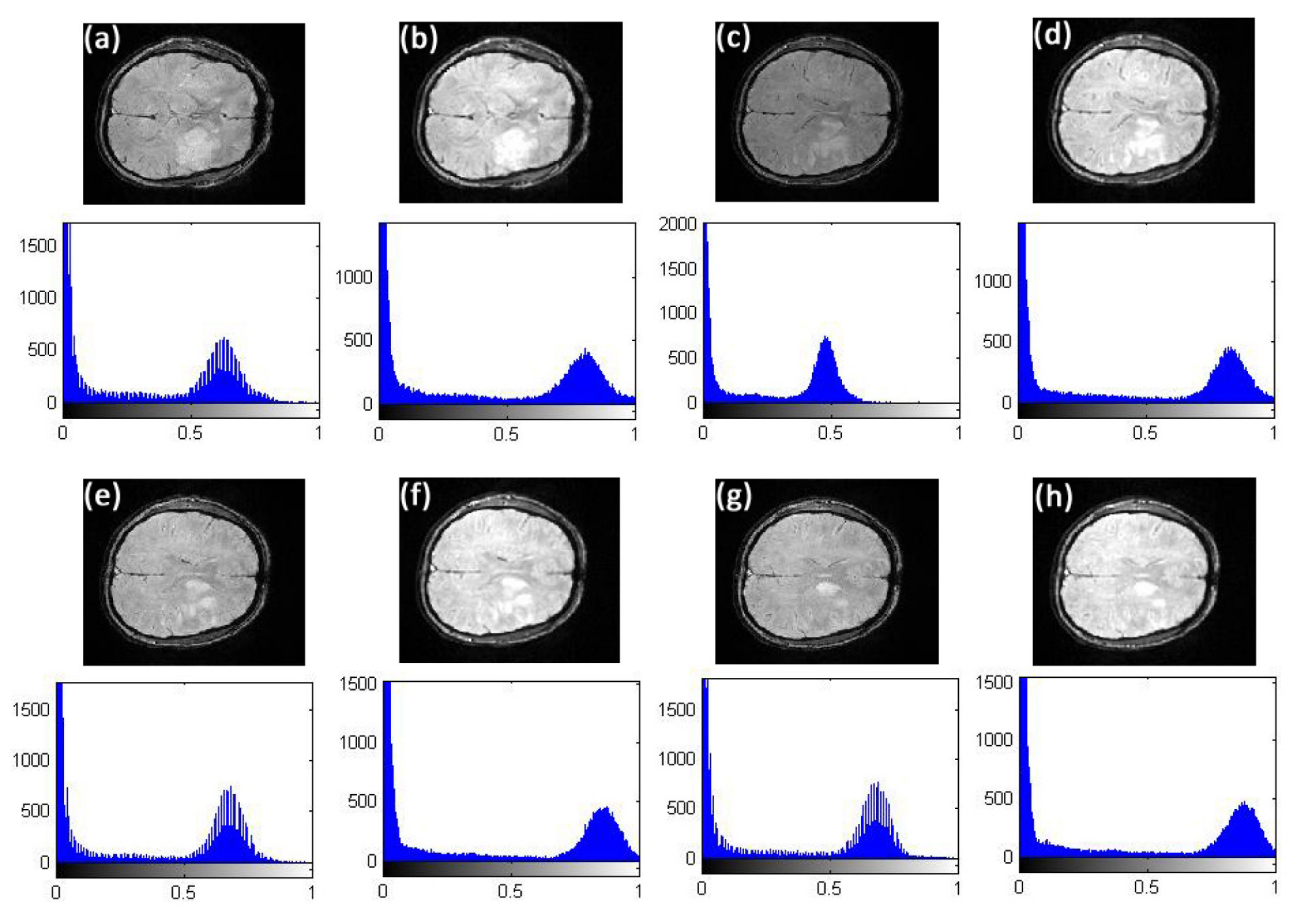
Comparison of texture details between original image slices 40, 44, 45, and 46 of first stroke patient and their fractional differential using VOFCD with a 5 × 5 mask using weights (0.45, 0.01, 0.54). (a) Original image slice 40 with histogram, (b) image slice 40 using VOFCD with histogram, (c) original image slice 44 with histogram, (d) image slice 44 using VOFCD with histogram, (e) original image slice 45 with histogram, (f) image slice 45 using VOFCD with histogram, (g) original image slice 46 with histogram, (h) image slice 46 using VOFCD with histogram.

**Fig 15:**
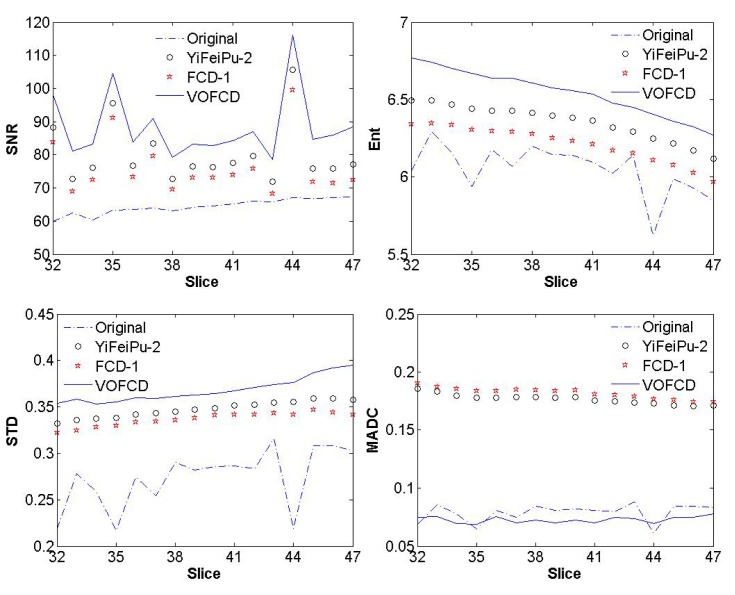
Comparison for SNR, Ent, STD and MADC between original image slices of first stroke patient and its fractional differential with mask 5 × 5 between VOFCD using weights (0.45, 0.01, 0.54) and YiFeiPU-2 and FCD-1 with *v* = 0.5.

Figs 2, 3, 14 and 15 provide the corresponding results obtained with (0.45, 0.01, 0.54). We now present clinical data used to test our methods on human brain images from first patient diagnosed with stroke. In Fig 2, we compare VOFCD with YiFeiPU-2 and FCD-1. Fig 3 shows the comparison of texture details in the region of interest between the original image slice 35 and its differential using the YiFeiPU-2, FCD-1 and VOFCD methods. It can be seen from Figs 2 and 3 that the image resulting from our method (VOFCD) is qualitatively better than the images constructed using the other methods. We note in particular that the visual effect offered by VOFCD is better than that of YiFeiPU-2 and FCD-1 with fractional order *v* = 0.5 when using the same mask dimensions. This choice of *v* allows us to make consistent comparisons across the different methods. Additional comparisons using different values of *v* are provided as Supporting Information.

Through the use of histograms, Fig 14 shows the comparison of texture details between the original image slices 40, 44, 45, and 46 of first stroke patient, and their fractional differential using VOFCD with a 5 × 5 mask. Again, we can see that VOFCD has enhanced the textural details in these images.

Fig 15 provides a comparison of SNR between the original image slices of first stroke patient and the fractional differential with mask 5 × 5 for VOFCD, YiFeiPU-2 and FCD-1 with *v* = 0.5. From Fig 15, it can be seen that VOFCD produces the largest values of SNR, which implies a superior texture enhancement over the other methods.

We now present clinical data to test our methods on a human brain image from a patient diagnosed with Parkinson’s disease prior to surgery.

**Fig 16:**
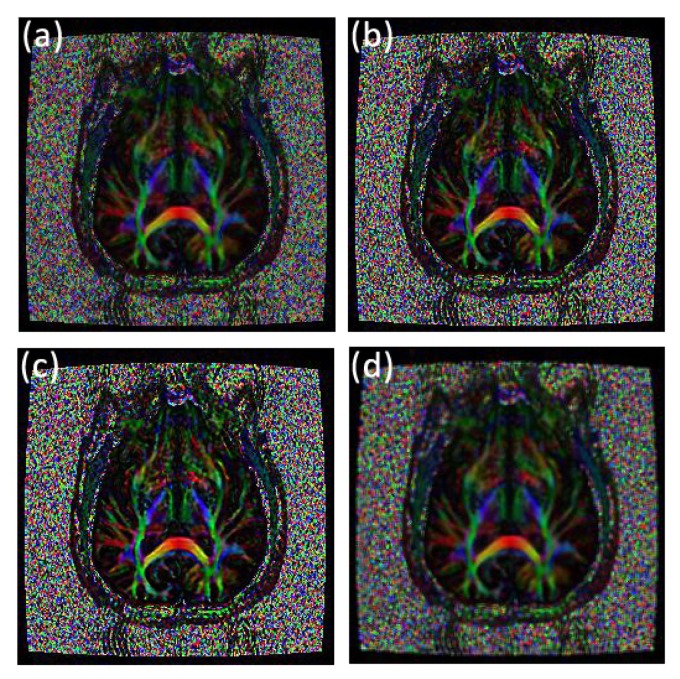
Comparison of texture details between an original fractional anisotropy weighted orientation map and its fractional differential using YiFeiPU-2, FCD-1 and VOFCD and (0.25, 0.74.0.01) for the weights. (a) Original image, (b) 0.5–order YiFeiPU-2 with mask 5 × 5, (c) 0.5–order FCD-1 with mask 5 × 5, (d) VOFCD with mask 5 × 5.

Applying the fractional differential to the three elements in the HSI colour space, respectively, and then reverting to RGB colour space, one can obtain a colour image without distortion. Fig 12 shows that the parameter set (0.25, 0.74.0.01) obtained the largest SNR for an original fractional anisotropy weighted orientation map from a patient diagnosed with Parkinson’s disease prior to surgery with mask 5 × 5, and the parameter set (0.01, 0.01, 0.98) produced the largest MADC. Fig 16 shows the comparison of texture details between an original fractional anisotropy weighted orientation map and its fractional differential using YiFeiPU-2, FCD-1 and VOFCD using weights (0.25, 0.74.0.01). Table 4 provides the comparison of SNR for a region of interest of a fractional anisotropy weighted colour orientation map between VOFCD using (0.25, 0.74.0.01) and YiFeiPU-2 and FCD-1 with *v* = 0.5. We can conclude from Table 4 that VOFCD has the largest SNR in comparison to the other fractional order differential methods. The value of this measure is consistent with the VOFCD image shown in Fig 16, wherein features appear to be smoother than in the other images.

**Table 4:** Comparison of SNR of region of interest of fractional anisotropy weighted orientation map in colour between VOFCD using weights (0.25, 0.74.0.01) and YiFeiPU-2 and FCD-1 with *v* = 0.5 and 5 × 5 mask.

Operators for comparison	SNR
YiFeiPU-2	0.3318
FCD-1	0.3167
VOFCD	0.5704

**Fig 17:**
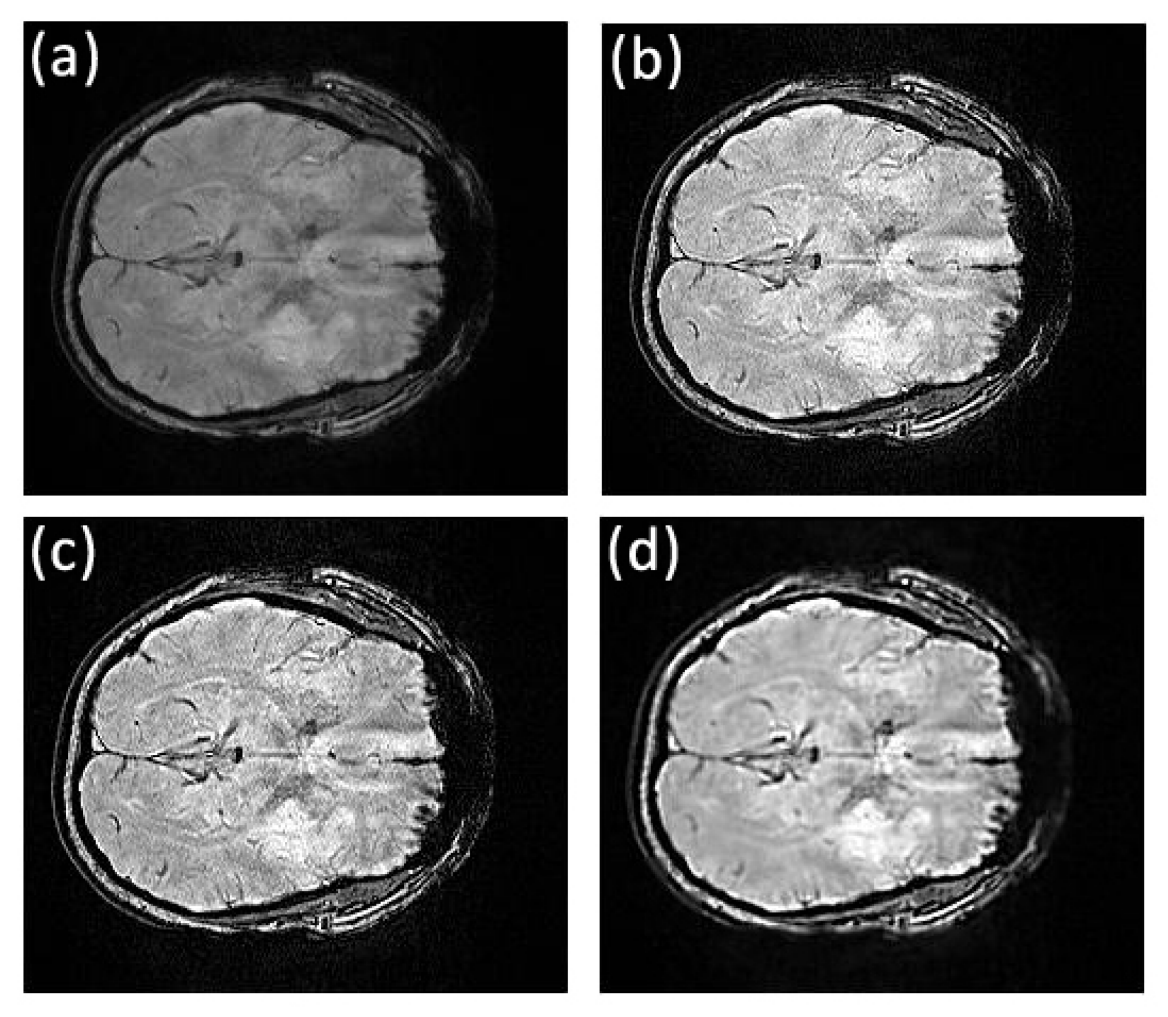
Comparison of texture details between original image slice 35 from the first stroke patient and its fractional differential using YiFeiPU-2, FCD-1 and VOFCD and (0.01, 0.01, 0.98) for the weights. (a) Original image slice 35, (b) 0.5–order YiFeiPU-2 with mask 5 × 5, (c) 0.5–order FCD-1 with mask 5 × 5, (d) VOFCD with mask 5 × 5.

**Fig 18:**
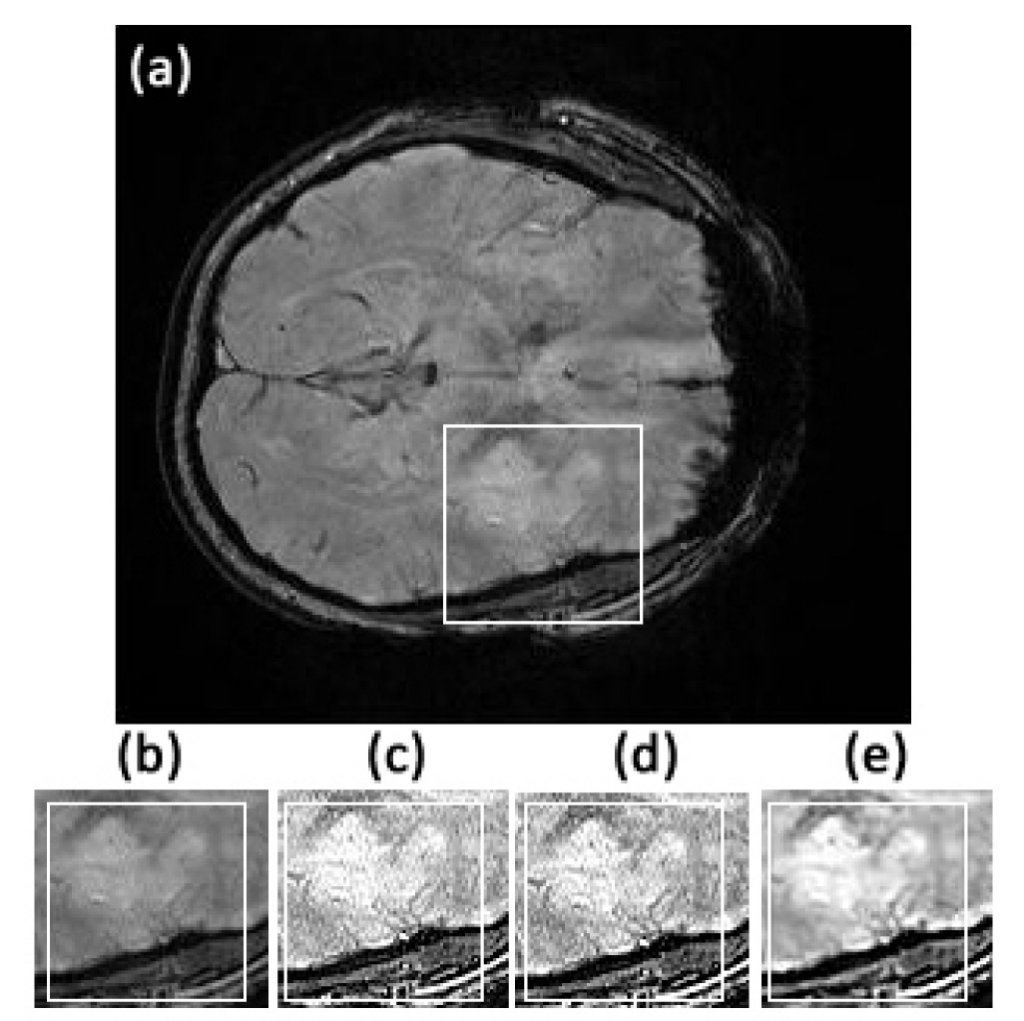
Comparison of texture details in the region of interest between original image slice 35 from the first stroke patient and its fractional differential using YiFeiPU-2, FCD-1 and VOFCD and (0.01, 0.01, 0.98) for the weights. (a) Original full image slice 35, (b) original region of interest, (c) 0.5–order YiFeiPU-2 with mask 5 × 5, (d) 0.5–order FCD-1 with mask 5 × 5, (e) VOFCD with mask 5 × 5.

**Fig 19:**
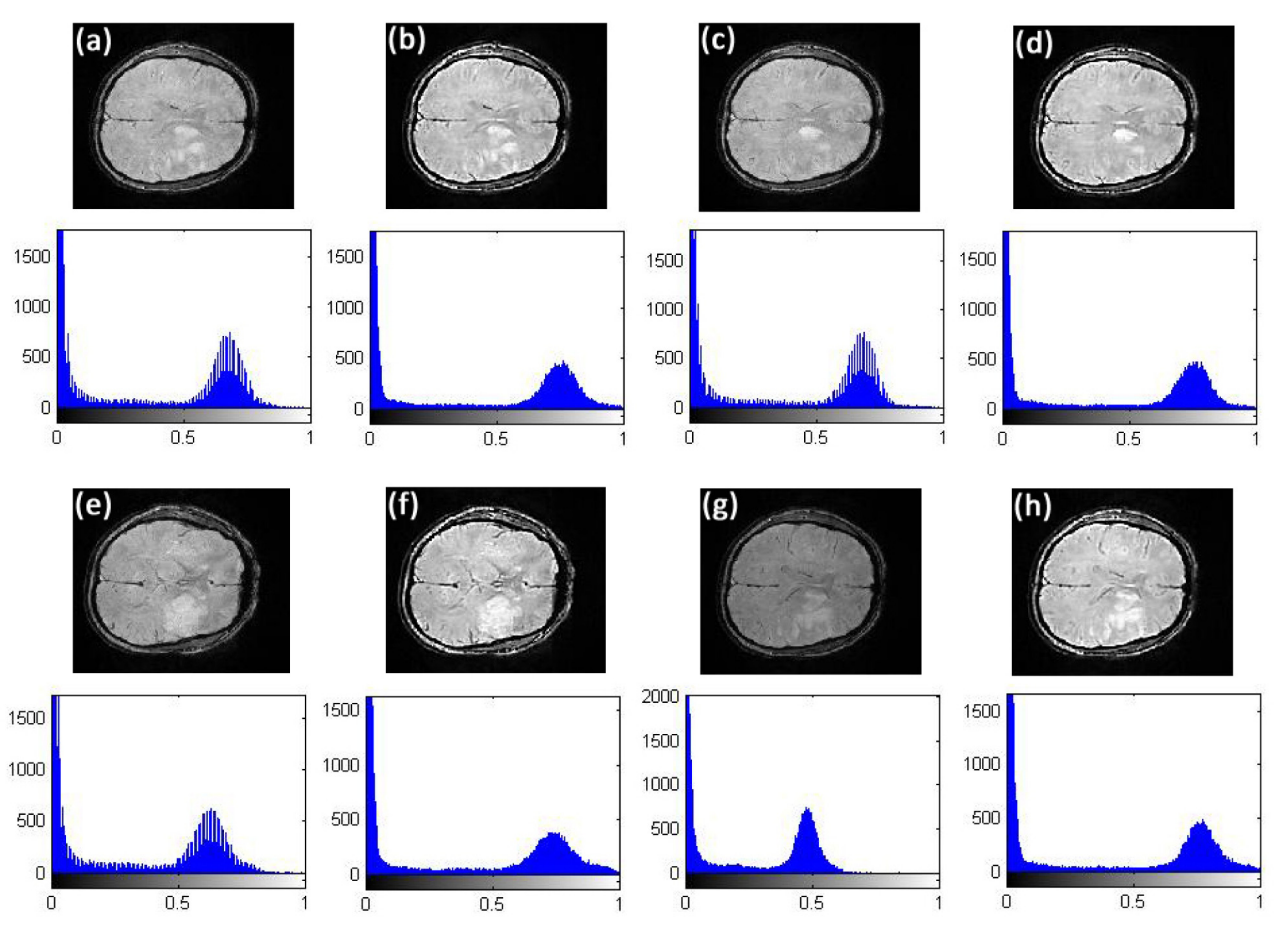
Comparison of texture details between original image slices 40, 44, 45, and 46 of first stroke patient and their fractional differential using VOFCD with a 5 × 5 mask using weights (0.01, 0.01, 0.98). (a) Original image slice 40 with histogram, (b) image slice 40 using VOFCD with histogram, (c) original image slice 44 with histogram, (d) image slice 44 using VOFCD with histogram, (e) original image slice 45 with histogram, (f) image slice 45 using VOFCD with histogram, (g) original image slice 46 with histogram, (h) image slice 46 using VOFCD with histogram.

**Fig 20:**
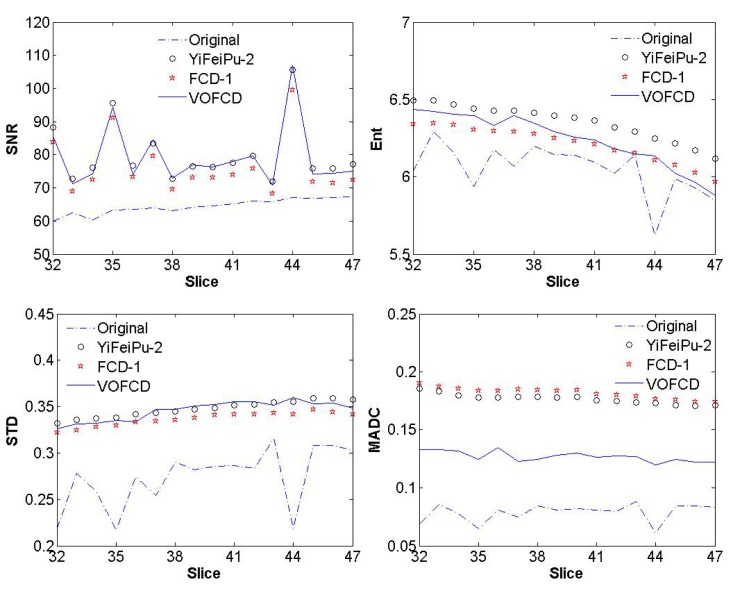
Comparison for SNR, Ent, STD and MADC between original image slices of first stroke patient and its fractional differential with mask 5 × 5 between VOFCD using weights (0.01, 0.01, 0.98) and YiFeiPU-2 and FCD-1 with *v* = 0.5.

**Fig 21:**
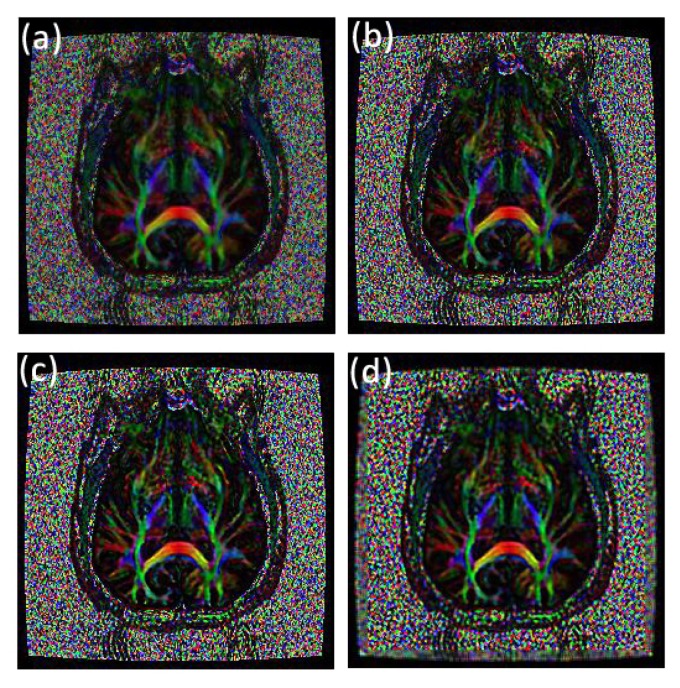
Comparison of texture details between an original fractional anisotropy weighted orientation map and its fractional differential using YiFeiPU-2, FCD-1 and VOFCD and (0.01, 0.01, 0.98) for the weights. (a) Original image, (b) 0.5–order YiFeiPU-2 with mask 5 × 5, (c) 0.5–order FCD-1 with mask 5 × 5, (d) VOFCD with mask 5 × 5.

In a similar manner to the first set of weights, Figs 17–21 and Table 5 show the results using weights (0.01, 0.01, 0.98). With this choice of weights, the SNR is reduced however MADC increases. This finding is consistent with a likely increase in image contrast. Hence, the choice of weights can be used to influence or trade-off SNR against contrast. It is unlikely that a fixed choice of weight can be applied to any image, since images not only vary in modality but also have different types of textures and contrast within them.

**Table 5:** Comparison of SNR of region of interest of fractional anisotropy weighted orientation map in colour between VOFCD using weights (0.01, 0.01, 0.98) and YiFeiPU-2 and FCD-1 with *v* = 0.5 and 5 × 5 mask.

Operators for comparison	SNR
YiFeiPU-2	0.3318
FCD-1	0.3167
VOFCD	0.3906

### Quantitative Analysis

Measures of Ent, STD and MADC were used for the quantitative analysis. Fig 15 provides the comparison of this analysis between the original image slices of first stroke patient and the fractional differential with mask 5 × 5 for VOFCD using weights (0.45, 0.01, 0.54), YiFeiPU-2 and FCD-1 with *v* = 0.5. It can be seen from Fig 15 that VOFCD has larger Ent values than the other fractional differential methods, and together with Fig 2 we can see that VOFCD can effectively enhance the image quality. The results presented in Fig 15 allow us to conclude that using VOFCD, the values of STD are larger than the other fractional differential methods. The increase in STD is due to increased signal intensities across the entire image. Furthermore, VOFCD has a smaller value of MADC than the other fractional differential methods, which implies a reduction in noise in VOFCD reconstructed images.

Based on a human brain image from a patient diagnosed with Parkinson’s disease prior to surgery, Table 6 provides a comparison of the relevant quantitative analysis for a region of interest of a fractional anisotropy weighted colour orientation map between VOFCD using weights (0.25, 0.74.0.01) and YiFeiPU-2 and FCD-1 with *v* = 0.5. We conclude from Table 6 that VOFCD has the largest Ent value, and smallest STD and MADC values in comparison to the other fractional order differential methods. Again, the values of these measures are consistent with the VOFCD image shown in Fig 16, wherein features appear to be smoother than in the other images.

**Table 6:** Comparison of quantitative analysis of region of interest of fractional anisotropy weighted orientation map in colour between VOFCD using weights (0.25, 0.74.0.01) and YiFeiPU-2 and FCD-1 with *v* = 0.5. A 5 × 5 mask was used to generate the results.

Operators for comparison	Ent	STD	MADC
YiFeiPU-2	6.2858	0.1536	0.1239
FCD-1	6.0358	0.1885	0.1617
VOFCD	6.1749	0.1612	0.1045

In a similar manner to the first set of weights, Fig 20 and Table 7 show the results using weights (0.01, 0.01, 0.98).

**Table 7:** Comparison of quantitative analysis of region of interest of fractional anisotropy weighted orientation map in colour between VOFCD using weights (0.01, 0.01, 0.98) and YiFeiPU-2 and FCD-1 with *v* = 0.5. A 5 × 5 mask was used to generate the results.

Operators for comparison	Ent	STD	MADC
YiFeiPU-2	6.2858	0.1536	0.1239
FCD-1	6.0358	0.1885	0.1617
VOFCD	6.3168	0.1696	0.1095

We have established that the use of the fractional differential not only nonlinearly preserves the contour features in smooth areas, but also maintains a high frequency edge feature in areas where the grey scale has obvious changes. It also preserves high frequency characteristics of texture detail in those areas where the grey scale does not change considerably. Furthermore, VOFCD has a higher SNR than the other fractional differential mask operators, and our quantitative analysis verified that VOFCD leads to superior texture enhancement.

Using our method it is possible to achieve different effects based on the choice of the weights used in the reconstruction (i.e.*k*_1_, *k*_2_ and *k*_3_). For example, we showed that SNR can be maximised through appropriate choices of the weights. Similarly, we found weights that maximised MADC. Therefore, the choices of the weights can be optimised for the application. Nonetheless, it is difficult to establish a set of weights that can be applied robustly across all applications, since particular applications may rely on suppression of noise (i.e. SNR improvements) and others on differentiation of structures within images (i.e. MADC improvements).

## Conclusion

For grey scale and colour image enhancement, we derived an improved fractional differential algorithm, VOFCD, based on the second order Riesz fractional differential operator using a Lagrange 3–point interpolation formula. We estimated the fractal dimension of a local region instead of using fixed fractional order differentiation. The experiments showed that the use of VOFCD results in higher SNR than the existing methods of YiFeiPU-2 and FCD-1, which implies superior texture enhancement of medical images. In addition, VOFCD produces qualitatively better results than YiFeiPU-2 and FCD-1 with variable fractional order and same mask dimensions. A quantitative analysis was conducted to verify that VOFCD produces superior texture enhancement in comparison to the other methods considered. This may be helpful in clinical diagnosis and monitoring, and as future work, further analysis of this data will be carried out in conjunction with medical specialists. The optimal choice of reasonable parameters to obtain the variable fractional differential order remains challenging. In future research, we plan to perform an evaluation of how the weighted parameters and regularisation parameters affect the performance of the variable fractional differential order algorithm.

## Supporting Information

S1 Fig Convergence curves for SNR, Ent, STD and MADC using a 3 × 3 mask for image slice 34 from the first stroke patient. (EPS)

S2 Fig Convergence curves for SNR, Ent, STD and MADC using a 5 × 5 mask for image slice 34 from the first stroke patient. (EPS)

S3 Fig Convergence curves for SNR, Ent, STD and MADC using a 7 × 7 mask for image slice 34 from the first stroke patient. (EPS)

S4 Fig Convergence curves for SNR, Ent, STD and MADC using a 3 × 3 mask for image slice 36 from the first stroke patient. (EPS)

S5 Fig Convergence curves for SNR, Ent, STD and MADC using a 5 × 5 mask for image slice 36 from the first stroke patient. (EPS)

S6 Fig Convergence curves for SNR, Ent, STD and MADC using a 7 × 7 mask for image slice 36 from the first stroke patient. (EPS)

S7 Fig Convergence curves for SNR, Ent, STD and MADC using a 3 × 3 mask for image slice 40 from the first stroke patient. (EPS)

S8 Fig Convergence curves for SNR, Ent, STD and MADC using a 5 × 5 mask for image slice 40 from the first stroke patient. (EPS)

S9 Fig Convergence curves for SNR, Ent, STD and MADC using a 7 × 7 mask for image slice 40 from the first stroke patient. (EPS)

S10 Fig Convergence curves for SNR, Ent, STD and MADC using a 3 × 3 mask for image slice 44 from the first stroke patient. (EPS)

S11 Fig Convergence curves for SNR, Ent, STD and MADC using a 5 × 5 mask for image slice 44 from the first stroke patient. (EPS)

S12 Fig Convergence curves for SNR, Ent, STD and MADC using a 7 × 7 mask for image slice 44 from the first stroke patient. (EPS)

S13 Fig Convergence curves for SNR, Ent, STD and MADC using a 3 × 3 mask for image slice 45 from the first stroke patient. (EPS)

S14 Fig Convergence curves for SNR, Ent, STD and MADC using a 5 × 5 mask for image slice 45 from the first stroke patient. (EPS)

S15 Fig Convergence curves for SNR, Ent, STD and MADC using a 7 × 7 mask for image slice 45 from the first stroke patient. (EPS)

S16 Fig Convergence curves for SNR, Ent, STD and MADC using a 3 × 3 mask for image slice 46 from the first stroke patient. (EPS)

S17 Fig Convergence curves for SNR, Ent, STD and MADC using a 5 × 5 mask for image slice 46 from the first stroke patient. (EPS)

S18 Fig Convergence curves for SNR, Ent, STD and MADC using a 7 × 7 mask for image slice 46 from the first stroke patient. (EPS)

S19 Fig Convergence curves for SNR, Ent, STD and MADC using a 3 × 3 mask for image slice 44 from the second stroke patient. (EPS)

S20 Fig Convergence curves for SNR, Ent, STD and MADC using a 5 × 5 mask for image slice 44 from the second stroke patient. (EPS)

S21 Fig Convergence curves for SNR, Ent, STD and MADC using a 7 × 7 mask for image slice 44 from the second stroke patient. (EPS)

S22 Fig Convergence curves for SNR, Ent, STD and MADC using a 3 × 3 mask for image slice 60 from the second stroke patient. (EPS)

S23 Fig Convergence curves for SNR, Ent, STD and MADC using a 5 × 5 mask for image slice 60 from the second stroke patient. (EPS)

S24 Fig Convergence curves for SNR, Ent, STD and MADC using a 7 × 7 mask for image slice 60 from the second stroke patient. (EPS)

S25 Fig Comparison for SNR, Ent, STD and MADC between original image slices of first stroke patient and its fractional differential with mask 5 × 5 between VOFCD using weights (0.45, 0.01, 0.54) and YiFeiPU-2 and FCD-1 with *v* = 0.1. (EPS)

S26 Fig Comparison for SNR, Ent, STD and MADC between original image slices of first stroke patient and its fractional differential with mask 5 × 5 between VOFCD using weights (0.45, 0.01, 0.54) and YiFeiPU-2 and FCD-1 with *v* = 0.3. (EPS)

S27 Fig Comparison for SNR, Ent, STD and MADC between original image slices of first stroke patient and its fractional differential with mask 5 × 5 between VOFCD using weights (0.45, 0.01, 0.54) and YiFeiPU-2 and FCD-1 with *v* = 0.7. (EPS)

S28 Fig Comparison for SNR, Ent, STD and MADC between original image slices of first stroke patient and its fractional differential with mask 5 × 5 between VOFCD using weights (0.45, 0.01, 0.54) and YiFeiPU-2 and FCD-1 with *v* = 0.9. (EPS)

S29 Fig Comparison for SNR, Ent, STD and MADC between original image slices of first stroke patient and its fractional differential with mask 5 × 5 between VOFCD using weights (0.01, 0.01, 0.98) and YiFeiPU-2 and FCD-1 with *v* = 0.1. (EPS)

S30 Fig Comparison for SNR, Ent, STD and MADC between original image slices of first stroke patient and its fractional differential with mask 5 × 5 between VOFCD using weights (0.01, 0.01, 0.98) and YiFeiPU-2 and FCD-1 with *v* = 0.3. (EPS)

S31 Fig Comparison for SNR, Ent, STD and MADC between original image slices of first stroke patient and its fractional differential with mask 5 × 5 between VOFCD using weights (0.01, 0.01, 0.98) and YiFeiPU-2 and FCD-1 with *v* = 0.7. (EPS)

S32 Fig Comparison for SNR, Ent, STD and MADC between original image slices of first stroke patient and its fractional differential with mask 5 × 5 between VOFCD using weights (0.01, 0.01, 0.98) and YiFeiPU-2 and FCD-1 with *v* = 0.9. (EPS)

S33 Fig Comparison of texture details between original image slice 51 from the second stroke patient and its fractional differential using YiFeiPU-2, FCD-1 and VOFCD and (0.35, 0.06, 0.59) for the weights. (a) Original image slice 51, (b) 0.5–order YiFeiPU-2 with mask 5 × 5, (c) 0.5–order FCD-1 with mask 5 × 5, (d) VOFCD with mask 5 × 5.
(EPS)

S34 Fig Comparison of textures in the region of interest between original image slice 51 from the second stroke patient and its fractional differential using YiFeiPU-2, FCD-1 and VOFCD and (0.35, 0.06, 0.59) for the weights. (a) Original full image slice 51, (b) original region of interest, (c) 0.5–order YiFeiPU-2 with mask 5 × 5, (d)0.5–order FCD-1 with mask 5 × 5, (e) VOFCD with mask 5 × 5.
(EPS)

S35 Fig Comparison of texture details between original image slice 51 from the second stroke patient and its fractional differential using YiFeiPU-2, FCD-1 and VOFCD and (0.01, 0.01, 0.98) for the weights. (a) Original image slice 51, (b) 0.5–order YiFeiPU-2 with mask 5 × 5, (c) 0.5–order FCD-1 with mask 5 × 5, (d) VOFCD with mask 5 × 5.
(EPS)

S36 Fig Comparison of textures in the region of interest between original image slice 51 from the second stroke patient and its fractional differential using YiFeiPU-2, FCD-1 and VOFCD and (0.01, 0.01, 0.98) for the weights. (a) Original full image slice 51, (b) original region of interest, (c) 0.5–order YiFeiPU-2 with mask 5 × 5, (d) 0.5–order FCD-1 with mask 5 × 5, (e) VOFCD with mask 5 × 5.
(EPS)
